# Efficient photocatalytic hydrogen peroxide generation coupled with selective benzylamine oxidation over defective ZrS_3_ nanobelts

**DOI:** 10.1038/s41467-021-22394-8

**Published:** 2021-04-01

**Authors:** Zhangliu Tian, Cheng Han, Yao Zhao, Wenrui Dai, Xu Lian, Yanan Wang, Yue Zheng, Yi Shi, Xuan Pan, Zhichao Huang, Hexing Li, Wei Chen

**Affiliations:** 1 SZU-NUS Collaborative Innovation Center for Optoelectronic Science & Technology, International Collaborative Laboratory of 2D Materials for Optoelectronics Science and Technology of Ministry of Education, Institute of Microscale Optoelectronics, Shenzhen University, Shenzhen, China; 2 Department of Chemistry, National University of Singapore, 3 Science Drive 3, Singapore, Singapore; 3 Joint School of National University of Singapore and Tianjin University, International Campus of Tianjin University, Binhai New City, Fuzhou, China; 4 Department of Physics, National University of Singapore, 2 Science Drive 3, Singapore, Singapore; 5 International Joint Lab on Resource Chemistry, College of Chemistry and Materials Science, Shanghai Normal University, Shanghai, China

**Keywords:** Artificial photosynthesis, Photocatalysis, Nanoscale materials

## Abstract

Photocatalytic hydrogen peroxide (H_2_O_2_) generation represents a promising approach for artificial photosynthesis. However, the sluggish half-reaction of water oxidation significantly limits the efficiency of H_2_O_2_ generation. Here, a benzylamine oxidation with more favorable thermodynamics is employed as the half-reaction to couple with H_2_O_2_ generation in water by using defective zirconium trisulfide (ZrS_3_) nanobelts as a photocatalyst. The ZrS_3_ nanobelts with disulfide (S_2_^2−^) and sulfide anion (S^2−^) vacancies exhibit an excellent photocatalytic performance for H_2_O_2_ generation and simultaneous oxidation of benzylamine to benzonitrile with a high selectivity of >99%. More importantly, the S_2_^2−^ and S^2−^ vacancies can be separately introduced into ZrS_3_ nanobelts in a controlled manner. The S_2_^2−^ vacancies are further revealed to facilitate the separation of photogenerated charge carriers. The S^2−^ vacancies can significantly improve the electron conduction, hole extraction, and kinetics of benzylamine oxidation. As a result, the use of defective ZrS_3_ nanobelts yields a high production rate of 78.1 ± 1.5 and 32.0 ± 1.2 μmol h^−1^ for H_2_O_2_ and benzonitrile, respectively, under a simulated sunlight irradiation.

## Abstract (web summary)

Photocatalytic H_2_O_2_ generation represents a promising approach for artificial photosynthesis. Here, ZrS_3_ nanobelts with controllable disulfide and sulfide anion vacancies exhibit an excellent photocatalytic H_2_O_2_ generation performance together with selective oxidation of benzylamine to benzonitrile.

## Introduction

Artificial photosynthesis, i. e. the conversion of solar energy into chemical energy, is considered as one of the promising approaches to synthesize chemicals with the unlimited energy source and minimized environmental problems^1,2^. As a promising liquid solar fuel generated by artificial photosynthesis, hydrogen peroxide (H_2_O_2_) has attracted growing attention because of its high commercial value and low transportation cost^3^. Substantial efforts have been devoted to the development of effective photocatalysts or photocathodes for the H_2_O_2_ generation from water and O_2_^4–6^. In the photocatalytic process, the sluggish oxidation of water induced by the photogenerated valence holes is a limiting factor for the production of H_2_O_2_^7–11^. Most previous reports focused on improving the half-reaction of O_2_ reduction, e. g. by consuming holes with sacrificial agents, such as isopropyl alcohol, benzyl alcohol, and 2-PrOH^3,12,13^. However, the development of an alternative oxidation reaction with accelerated kinetics to produce value-added chemicals was rarely reported. On the other hand, selective oxidation of amines to nitriles with lower oxidation potential than water plays an vital role in both laboratorial and industrial synthetic process since nitriles are the important intermediates during the synthesis of fine chemicals, pharmaceuticals, and agrochemicals^14–21^. Intensive research has been carried out to synthesize nitriles from primary amines through dehydrogenation^22–28^. However, most of the reactions are conducted in organic solvents under harsh conditions, such as high-temperature, exposure to high-pressure oxygen or air, and presence of oxidants. Photocatalytic reactions have been demonstrated to be an effective approach to synthesize nitriles under mild conditions^29–31^, but previous works were seriously limited by the use of noble metals as the co-catalysts to realize the dehydrogenation in organic solvents. Thus, the development of artificial photosynthesis in an aqueous and easy scale-up condition with earth-abundant photocatalysts is highly desirable for the production of nitriles in an economically-viable and environment-friendly way.

Monoclinic zirconium trisulfide (ZrS_3_) (ICCD PDF no. 30-1498), a layered n-type transition metal trichalcogenide (TMT), has recently drawn great research interest due to the extraordinary properties arising from its unique disulfide anions (S_2_^2−^)^32^. ZrS_3_ has shown a good optical responsivity of 290 mA W^−1^ with an in-plane hole and electron mobility at a magnitude of 10^2^ and 10^3^ cm^2^ V^−1^ s^−1^, respectively^33–36^. In particular, ZrS_3_ possesses a bandgap of ~2 eV with a more negative conduction band minimum (CBM) than the H_2_ evolution potential^37^, making ZrS_3_ a promising semiconductor for photocatalytic and photoelectrochemical applications. The previous studies on zirconium nitride have demonstrated that its superior performance for O_2_ reduction stems from the interaction between Zr sites and oxide species, where the Zr d-orbitals make a strong contribution^38^. Intriguingly, the conduction band of ZrS_3_ is mainly composed of Zr d-orbitals^33,35^, which has a much more negative potential than the reducing potential of O_2_ to H_2_O_2_. Therefore, ZrS_3_ shows great potential for the photocatalytic H_2_O_2_ generation.

**Fig. 1:**
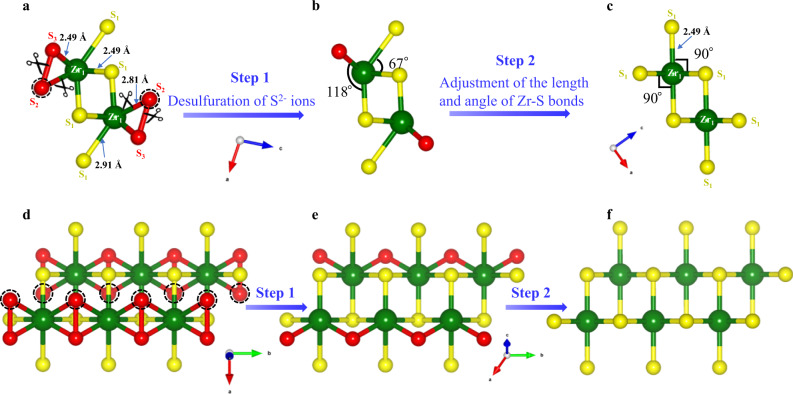
The transformation of the crystal structure of ZrS_3_ into ZrS_2_. The schematic process of the transformation of monoclinic ZrS_3_ (ICCD PDF no. 30-1498) into hexagonal ZrS_2_ (ICCD PDF no. 11-0679) from the [010] (**a**–**c**) and [001] (**d**–**f**) views. **a**, **d** Crystal structure of monolayer ZrS_3_ with a boundary of 1 x 3 x 1 from the [010] and [001] views, respectively. **b**, **e** Crystal structure of monolayer ZrS_3_ after desulfuration of S_2_^2−^ ions from **a** and **d**, respectively. **c**, **f** Crystal structure of monolayer ZrS_2_ with a boundary of 1 x 3 x 1 from the [010] and [001] views, respectively.

There are three categories of S (S_1_, S_2_, and S_3_) environments in monoclinic ZrS_3_ lattice, where S_1_ denotes the sulfide ion (S^2−^) and S_2_, S_3_ are interpreted as the S_2_^2−^ (Fig. 1a, b)^39^. Recently, both theoretical calculations and experimental investigations have demonstrated that moderate S_2_^2−^ vacancies can greatly promote the separation of photogenerated charge carriers in TMTs (Supplementary Fig. 1a and b)^40,41^. In addition, the anion vacancies existing on the surface of n-type semiconductors can further improve its photocatalytic and photoelectrochemical performance by accelerating the kinetics of hole transfer on the surface^42–45^. The crystal structure analysis of ZrS_3_ inspires us that the S_2_^2−^ and S^2−^ vacancies can be separately introduced into ZrS_3_ by different methods (Fig. 1 and Supplementary Fig. 1). Experimentally, hexagonal ZrS_2_ (ICCD PDF no. 11-0679) is usually obtained by vacuum annealing of monoclinic ZrS_3_ at elevated temperatures. This suggests that ZrS_3_ can desulfurize into ZrS_2_ by the post-annealing at a higher temperature under vacuum^46^. Only one type of Zr (Zr_1_) and S (S_1_) environment exists in ZrS_2_, where the Zr_1_-S_1_ bond length is similar to that in ZrS_3_ (Fig. 1a–c). Besides, ZrS_2_ shows a similar layered structure to ZrS_3_, and atomic layers in both materials are parallel to the (001) plane (Supplementary Fig. 1c–f). When ZrS_3_ transforms into ZrS_2_, it does not need much tweaking of the framework along both [010] and [001] directions (Supplementary Fig. 1c and e), but it is required to adjust the framework along the [100] direction (Fig. 1d and f). Figure 1 clearly suggests that the ZrS_3_ can transform into ZrS_2_ by two steps: the first step can be the desulfurization of ZrS_3_ to release S_2_ or S_3_ ions to form a distorted crystal structure of ZrS_2_ (Fig. 1a, b, d, and e), and then the distorted crystal structure undergoes structural relaxation by tuning the length and angle of Zr-S bonds to form ZrS_2_ without breaking or regrouping the bonds (Fig. 1b, c, e, and f). Thus, the high-temperature vacuum annealing is expected to be an effective scheme to produce S_2_^2−^ vacancies in ZrS_3_. On the other hand, S^2−^ ions have high adaptability when coordinated with metal ions, which can serve as either terminal or bridge ions to interact with metals (especially for alkali metals). This different from the S_x_^2−^ (x ≥ 2) ions that are difficult to bond with metals (Supplementary Fig. 1g)^47^. Moreover, ZrS_3_ is easily formed as nanobelts (NBs) with rich S^2−^ ions exposed at the edges (Supplementary Fig. 1h and i). Previous studies have used active metals such as Mg, Al, and Zn to induce oxygen vacancies in metal oxide due to their reducibility^48^. Compared to these metals, alkali metal lithium (Li) has a higher reducibility and can be easily intercalated into host materials. Li can be easily dissolved in solvents like ammonia or ethanediamine to form Li-based complex for the solvothermal treatment, which has been widely utilized to enhance the transition temperature of superconducting materials^49–51^ and in particular to induce oxygen vacancies on TiO_2_^52^. Therefore, such Li-based treatment could be an effective approach to induce S^2−^ vacancies on ZrS_3_.

Here, ZrS_3_ NBs with both S_2_^2−^ and S^2−^ vacancies are employed to enhance the photocatalytic production of H_2_O_2_ coupled with the selective oxidation of benzylamine to benzonitrile in water. The impacts of S_2_^2−^ and S^2−^ vacancies on modulating the charge carrier dynamics and photocatalytic performance are systematically investigated. The S_2_^2−^ vacancies can significantly facilitate the separation of photogenerated charge carriers; while the S^2−^ vacancies are demonstrated to not only promote the electron conduction and hole extraction in the photocatalytic process but also improve the kinetics of benzylamine oxidation. As a result, the use of defective ZrS_3_ NBs as photocatalyst produces H_2_O_2_ and benzonitrile at a high rate of 78.1 ± 1.5 and 32.0 ± 1.2 μmol h^−1^ respectively, under the illumination of a simulated sunlight.

## Results and discussions

### Structural properties and band structures of photocatalysts

**Fig. 2:**
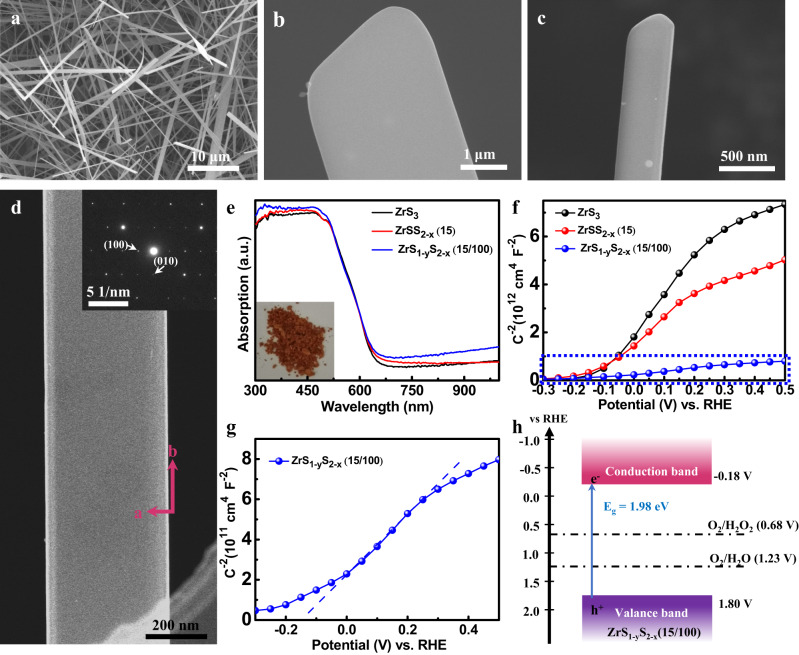
Structural properties and band structures of defective ZrS_3_ NBs. **a** Top-sectional, **b**, **c** high-magnification SEM images of the ZrS_3_ NBs. **d** TEM image and SAED pattern of single ZrS_3_ NB. **e** Diffuse reflectance UV–vis spectra of the ZrS_3_, ZrSS_2-x_(15) and ZrS_1-y_S_2-x_(15/100) NBs. Inset, the photograph of the ZrS_1-y_S_2-x_(15/100) NBs. **f** Mott–Schottky plots of ZrS_3_, ZrSS_2-x_(15) and ZrS_1-y_S_2-x_(15/100) NBs and **g** Mott-Schottky plot of ZrS_1-y_S_2-x_(15/100) magnified from **f**. **h** Schematic band structure diagram for ZrS_1-y_S_2-x_(15/100). To have a clear view of the single NB, the sample for this SEM measurement was prepared by evaporating the isopropanol dispersion of ZrS_3_ NBs.

ZrS_3_ NBs were synthesized via a chemical vapor transport of S powder to Zr powder using iodine as a transport agent. ZrS_3_ with S_2_^2−^ vacancies (ZrSS_2-x_) was obtained by the re-annealing of the as-grown ZrS_3_ NBs at 700 °C for different time (10, 15, and 20 min) under vacuum. ZrSS_2-x_ with S^2−^ vacancies (ZrS_1-y_S_2-x_) was prepared through a low-temperature solvothermal treatment by using Li-dissolved ethanediamine with different amounts of Li (50, 100, and 150 mg). We denote ZrSS_2-x_ NBs annealed for X time as ZrSS_2-x_(X) and ZrS_1-y_S_2-x_ NBs annealed for X min and treated with Y mg Li as ZrS_1-y_S_2-x_(X/Y). The x-ray diffraction (XRD) pattern indicates the formation of ZrS_3_ in the monoclinic phase (ICCD PDF no. 30-1498), and the vacuum annealing and further Li treatment did not induce any phase transition in ZrSS_2-x_(15) and ZrS_1-y_S_2-x_(15/100) NBs (Supplementary Fig. 2). However, the samples show decreased peak intensity from the ZrS_3_ to the ZrS_1-y_S_2-x_(15/100) NBs, due to the introduction of sulfur vacancies that reduce the crystallinity of ZrS_3_, as observed in the high-resolution transmission electron microscopy (HRTEM) image (Supplementary Fig. 3). The obtained ZrS_3_ was formed as NBs with the width ranging from 300 nm to 3 μm and length in tens of micrometers (Fig. 2a–c). Scanning electron microscope (SEM) and atomic force microscope (AFM) measurements were further conducted to statistically determine the length, width, and thickness distribution of ZrS_3_ NBs. The average length, width, and thickness of NBs were measured to be 24 μm, 840 nm, and 38 nm, respectively (Supplementary Figs. 4 and 5), where all the histograms exhibit a unimodal distribution with the peak in the range of 20–30 μm, 0.6–1.0 μm and 25–45 nm, respectively (Supplementary Fig. 4b, c and Supplementary Fig. 5d). As a result, the average ratio of width/thickness was calculated to be ~22, which qualifies the label of “NBs” for our samples. The individual ZrS_3_ NB is confirmed as the single crystal along [010] direction by the TEM and corresponding selected area electron diffraction (SAED) characterization (Fig. 2d). It is demonstrated that the ZrS_3_ layer is parallel to the axial direction of NB, which is in favor of charge carrier transport^40^. As shown in the HRTEM images (Supplementary Fig. 3), ZrS_3_ exhibits highly-ordered lattice fringes with an excellent crystallinity, while ZrS_1-y_S_2-x_(15/100) shows an obvious lattice disorder with relatively poor crystallinity. This suggests that the introduction of sulfur vacancies could lead to a decreased crystallinity in ZrS_3_, consistent with the XRD results.

As shown in the diffuse reflectance UV–vis spectra (Fig. 2e), both ZrS_3_ and ZrSS_2-x_ (15) NBs absorb light with the wavelength up to ~650 nm, corresponding to a bandgap of 2.02 eV (Supplementary Fig. 6). ZrS_1-y_S_2-x_(15/100) NBs present a slight red-shift of absorption spectrum, revealing a smaller bandgap of 1.98 eV. The Mott−Schottky plots for all three samples exhibit positive slopes, indicating the n-type behavior of ZrS_3_ (Fig. 2f). These results were obtained by measuring the photocatalysts deposited on the fluorine-doped tin oxide (FTO) substrate. It is worth noting that the deposition process did not induce any obvious change of the photocatalyst (Supplementary Fig. 7a–c), suggesting that the sample on the FTO substrate measured with the photoelectrochemical set-up is essentially the same photocatalyst. The flat band potentials (*E*_fb_) of ZrS_3_, ZrSS_2-x_(15), and ZrS_1-y_S_2-x_(15/100) are estimated to be −0.10, −0.11, and −0.18 V *versus* reversible hydrogen electrode (*V*_RHE_), respectively (Fig. 2g and Supplementary Fig. 7d). *E*_fb_ is commonly used to estimate the CBM for a series of n-type semiconductors at the surface in an aqueous environment, which agreed with their theoretically determined values^1,44,53–55^. Previous studies on the energy positions of semiconductors have shown that the CBM of zirconium-based sulfides is very close to their *E*_fb_^37,54^, and therefore the CBM of ZrS_3_, ZrSS_2-x_(15), and ZrS_1-y_S_2-x_(15/100) can be directly determined by their *E*_fb_. Based on the Mott−Schottky (Fig. 2f, g, and Supplementary Fig. 7d) and UV–vis spectra results, the CBM and valance band maximum (VBM) for ZrS_3_, ZrSS_2-x_(15), and ZrS_1-y_S_2-x_(15/100) were revealed to be −0.10, −0.11, −0.18 *V*_RHE_ (CBM) and 1.92, 1.91, and 1.80 *V*_RHE_ (VBM), respectively (Fig. 2h and Supplementary Fig. 8). The CBMs of ZrS_3_, ZrSS_2-x_(15), and ZrS_1-y_S_2-x_(15/100) are higher than the potential for two-electron reduction of O_2_. Previous studies have shown that the oxidation potential of benzylamine lies higher than that of water, and the benzylamine oxidation was thus used to replace oxygen evolution reaction to couple with photocatalytic and electrocatalytic hydrogen evolution reaction^14,17,55,56^. This suggests the VBM of defective ZrS_3_ NBs lying far below the oxidation potential of benzylamine, indicating that these photocatalysts are applicable to the photocatalytic O_2_ reduction and benzylamine oxidation.

### Characterizations of vacancy structure

**Fig. 3:**
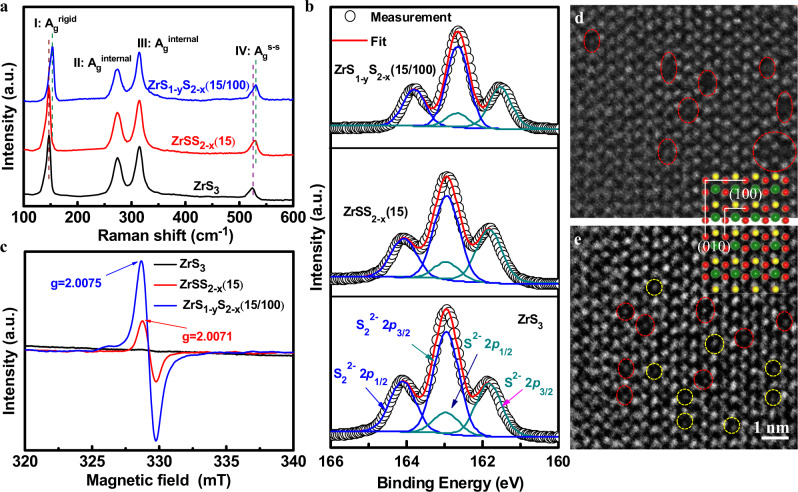
Characterizations of vacancy structure of defective ZrS_3_ NBs. **a** Raman spectra, **b** S 2*p* XPS spectra, and **c** EPR spectra of the ZrS_3_, ZrSS_2-x_(15) and ZrS_1-y_S_2-x_(15/100) NBs. HAADF-STEM images of **d** ZrSS_2-x_(15) and **e** ZrS_1-y_S_2-x_(15/100) measured from a spherical aberration-corrected TEM. Inset: the crystal lattice of ZrS_3_ along the [001] orientation. The red and yellow circles represent S_2_^2−^and S^2−^, respectively.

Four characteristic Raman modes of ZrS_3_ located at ~ 147, 274, 315, and 524 cm^−1^ were observed in Fig. 3a, which are assigned to the rigid chain vibration (I: A_g_^rigid^), internal out-of-plane vibrations (II: A_g_^internal^ and III: A_g_^internal^), and S–S diatomic motion (IV: A_g_^s–s^), respectively^35^. The Raman spectra show an obvious red-shift of A_g_^s–s^ mode by ~5 cm^−1^ from ZrS_3_, ZrSS_2-x_(15), and ZrS_1-y_S_2-x_(15/100), originating from the introduction of S_2_^2−^ vacancies^40^. We also observed a ~3 cm^−1^ red-shift of A_g_^rigid^ mode from ZrS_3_ and ZrSS_2-x_(15) to ZrS_1-y_S_2-x_(15/100). Since the A_g_^rigid^ is correlated to the vibration of quasi-one-dimensional chains in the direction of c axis (Supplementary Fig. 9a), the shift of A_g_^rigid^ mode in ZrS_1-y_S_2-x_(15/100) results from the introduction of S^2−^ vacancies, which alters the length of Zr–S bonds within each chain. The similar shift of A_g_^rigid^ mode was also identified from ZrS_3_ and ZrSS_2-x_(15) to the only Li-treated ZrS_3_ with 100 mg Li (ZrS_1-y_S(100)) as shown in Supplementary Fig. 9b. To explore the effect of process parameters during the synthesis of defective materials on the types and density of defects, and the correlation with photocatalytic activity, orthogonal experiments have been performed by simultaneously changing the Li amount and vacuum annealing duration. All the samples were further examined by the Raman characterization, and Supplementary Fig. 10a–d show representative Raman spectra of the defective ZrS_3_ NBs separately treated by the Li-treatment with different Li amount and by the vacuum annealing for different time. The gradual red-shift of A_g_^rigid^ mode (difference from 1.6 to 6.1 cm^−1^) was observed with increasing Li amount, resulting from the increased concentration of S^2−^ vacancies in Li-treated ZrS_3_ NBs (Supplementary Fig. 10a and b). Similarly, the vacuum annealing triggered a red-shift of A_g_^s-s^ mode due to the generated S_2_^2−^ vacancies^40^, which was enlarged from 2.4 to 8.1 cm^−1^ by prolonging the annealing time (Supplementary Fig. 10c and d). Based on the Raman results, the shifts of both A_g_^rigid^ and A_g_^s-s^ modes for all the samples were extracted and plotted as 3D histograms, as shown in Supplementary Figure 11a and b. The A_g_^rigid^ shift only depends on the Li amount, while the A_g_^s-s^ shift only relies on the annealing time. These results reveal that the S_2_^2−^ and S^2−^ vacancies in ZrS_1-y_S_2-x_ can be independently induced by the vacuum annealing and Li-treatment respectively, and the concentration of vacancies can be further controlled by varying the annealing time and Li amount.

The XPS characterization was conducted on these samples to further confirm the vacancy type. After the vacuum annealing, the ZrSS_2-x_(15) NBs exhibit a slightly lower binding energy of the Zr 3*d* core level than ZrS_3_ NBs, consistent with the results from ZrS_3_ to ZrS_2_ (Supplementary Fig. 12a)^57^. Furthermore, ZrSS_2-x_(15) shows a significant attenuation of S_2_^2−^ 2*p* peaks with the nearly unchanged S^2−^ 2*p* peaks compared to ZrS_3_ (Fig. 3b and Supplementary Fig. 12b), indicating the mere increase of S_2_^2−^ vacancies in ZrSS_2-x_(15). It is worth noting that no clear peak shift was observed for ZrSS_2-x_(15), which results from the almost retained electron density around the S sites, as revealed by the Mott-Schotty results. After further Li treatment, both Zr 3*d* and S 2*p* core levels of ZrS_1-y_S_2-x_(15/100) NBs shifted to the lower binding energy by ~0.3 eV regarding ZrSS_2-x_(15) (Supplementary Fig. 12a, c, and d), due to the increased electron density around the S sites induced by S^2−^ vacancies^58^. In particular, the intensity of S^2−^ 2*p* peaks in the ZrS_1-y_S_2-x_(15/100) was clearly lower than that of ZrSS_2-x_(15) (Fig. 3b and Supplementary Fig. 12d), revealing the increase of S^2−^ vacancies. This phenomenon has been commonly observed in the transition metal sulfide with S^2−^ vacancies such as MoS_2_, In_2_S_3_, and CuInS_2_^59–61^. The similar variation of Zr 3*d* and S 2*p* spectra observed from ZrS_3_ to ZrS_1-y_S(100) further suggest the separate introduction of S^2−^ vacancies by the Li treatment (Supplementary Fig. 12a and c). The type and density of sulfur vacancies for all the samples were further quantitatively analyzed by the XPS characterization, and representative S 2*p* XPS spectra of the defective ZrS_3_ NBs separately treated by the vacuum annealing and Li-treatment are presented in Supplementary Fig. 13a and b, respectively. The x and y values in the label of ZrS_1-y_S_2-x_ for all the samples were estimated from the XPS results by calculating the area ratio of characteristic peaks in defective samples to that of ZrS_3_, as summarized in Supplementary Table 1. Agreed with the Raman results, the vacuum annealing and Li-treatment can independently attenuate the intensity of S_2_^2−^ 2*p* and S^2−^ 2*p* peaks, respectively, as revealed by the almost unchanged x and y values under the identical annealing time and Li amount, respectively. For an intuitive comparison, the x and y values as a function of the annealing time and Li amount were plotted in 3D histograms, as shown in Supplementary Fig. 14a and b, respectively. The x was estimated to be 0.20 ± 0.01, 0.36 ± 0.01, and 0.49 ± 0.01 for the annealing time of 10, 15, and 20 min, respectively; while the y was evaluated to be 0.05 ± 0.01, 0.36 ± 0.01, and 0.49 ± 0.01 for 50, 100, and 150 mg Li, respectively.

In addition, the electron paramagnetic resonances (EPR) investigation was also carried out to detect the vacancy structure. A characteristic peak can be clearly detected at g = 2.0071 for all the vacuum annealed samples in Supplementary Fig. 15a, and the peak intensity is proportional to the vacuum annealing duration. This suggested the characteristic peak of Zr-S_2_^2−^ dangling bonds is located at g = 2.0071^58^. Similarly, the characteristic peak of Zr-S^2−^ dangling bonds is located at g = 2.0082 (Supplementary Fig. 15b). Therefore, the characteristic peak located at g = 2.0075 for ZrS_1-y_S_2-x_(15/100) NBs suggests the formation of both S^2−^ and S_2_^2−^ vacancies (Fig. 3c). The higher signal intensity of ZrS_1-y_S_2-x_(15/100) than that of ZrSS_2-x_(15) indicates more sulfur vacancies existing in ZrS_1-y_S_2-x_(15/100) NBs^60^. To have a direct view of the atomic arrangement for ZrSS_2-x_(15) and ZrS_1-y_S_2-x_(15/100) NBs, the high-angle annular dark-field scanning transmission electron microscopy (HAADF-STEM) images were obtained, where the atomic sites can be determined by comparing the HAADF-STEM image with the crystal structure of ZrS_3_ lattice along the [001] direction (Supplementary Fig. 15c and d). The ZrSS_2-x_(15) demonstrates the missing atoms only emerging on the S_2_^2−^ sites, as indicated by the red dashed circles in Fig. 3d, while the atomic vacancies exist on both S^2−^ (indicated by yellow dashed circles) and S_2_^2−^ sites for ZrS_1-y_S_2-x_(15/100) (Fig. 3e).

### Photocatalytic performance

**Fig. 4:**
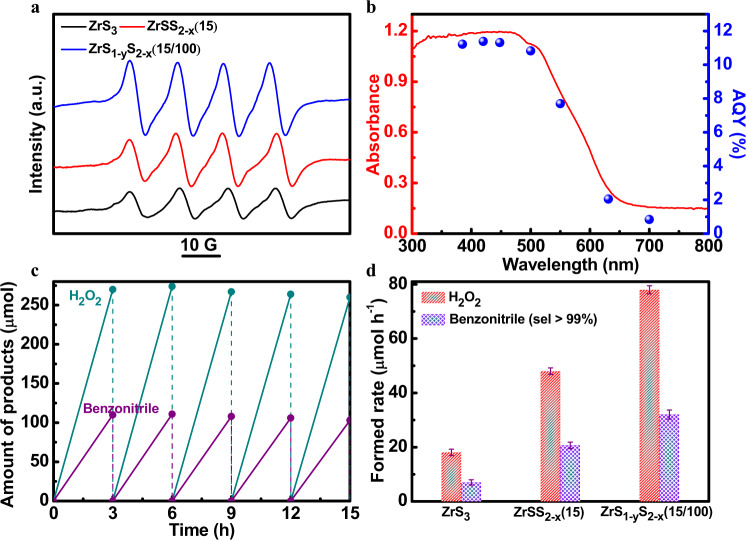
The photocatalytic properties of defective ZrS_3_ NBs. **a** EPR spectra of ZrS_3_, ZrSS_2-x_(15) and ZrS_1-y_S_2-x_(15/100) in the presence of DMPO. **b** Absorption spectrum of ZrS_1-y_S_2-x_(15/100) and its dependence of AQY with monochromatic light irradiation. Conditions: 30 ml aqueous solution with 1 mmol benzyl alcohol, 50 mg photocatalysts. **c** Results of H_2_O_2_ and benzonitrile generation for a repeated photoreaction sequence with ZrS_1-y_S_2-x_(15/100) under AM1.5G simulated sunlight irradiation. **d** H_2_O_2_ and benzonitrile evolution rate by the respective photocatalysts under AM1.5G simulated sunlight irradiation. Error bars are the standard error of the mean for 9 independent samples. Conditions: 30 ml H_2_O with 1 mmol benzylamine, 50 mg photocatalysts, 1 bar O_2_.

The photocatalytic capability of the defective ZrS_3_ NBs for reducing O_2_ to create the reactive oxygen species (ROS) was first evaluated by the EPR trapping experiment using 5,5-dimethyl-1-pyrroline N-oxide (DMPO). As illustrated in Fig. 4a, four characteristic peaks of DMPO−O_2_^•−^ were observed for all NBs, confirming the generation of O_2_^•−^^60,62,63^. The introduction of S_2_^2−^ vacancies was found to enhance the reduction of O_2_ to O_2_^•−^, and the additional introduction of S^2−^ vacancies led to a further increased photocatalytic activity. The correlation of types and density of sulfur vacancies with photocatalytic activity was further examined by the iodometry^12^ under the irradiation of AM1.5G simulated sunlight with the presence of benzyl alcohol as the hole scavenger. As shown in Supplementary Figure 16, the ZrS_1-y_S_2-x_(15/100) NBs with the x = 0.36 and y = 0.13 (16.3% sulfur vacancies) exhibit the best performance for photocatalytic H_2_O_2_ generation. When the Li amount and annealing time were simultaneously less than 150 mg and 20 min, respectively, the photocatalytic activity increased with the increase of both S_2_^2−^ and S^2−^ vacancies. When the Li amount reached 150 mg or the annealing time reached 20 min, the photocatalytic activity showed an increase at the early stage and then decreased with the increase of annealing time or Li amount, respectively. This indicates that it is harmful to further improve photocatalytic activity with excessive either S_2_^2−^ or S^2−^ vacancies. This is because excessive sulfur vacancies could act as the recombination centers for photogenerated charge carriers for photogenerated charge carriers. To determine the H_2_O_2_ formed rate, the production was analyzed by iodometry (Supplementary Fig. 17)^12^. The ZrS_1-y_S_2-x_(15/100) NBs possess a high H_2_O_2_ evolution rate of 89.6 ± 1.5 μmol h^−1^ with good reproducibility (Supplementary Fig. 18) in the presence of benzyl alcohol as the hole scavenger (entry 5 in Supplementary Table 2), which is higher than most previous reports (Supplementary Table 3). The wavelength-dependent apparent quantum yield (AQY) for the H_2_O_2_ generation on ZrS_1-y_S_2-x_(15/100) agrees well with its absorption spectrum, revealing that the photocatalytic activity originates from the bandgap excitation of ZrS_1-y_S_2-x_(15/100) (Fig. 4b). In particular, ZrS_1-y_S_2-x_(15/100) produces an AQY of 11.4 and 10.8% for the incident light of 400 and 500 nm respectively and demonstrates a good activity even with the excitation extended to the near-infrared region of ~700 nm. Furthermore, the photocatalyst of ZrS_1-y_S_2-x_(15/100) is able to maintain its activity after being recycled for the same reaction with both presence of benzylamine and benzyl alcohol, as presented in Fig. 4c and Supplementary Fig. 19a. After the stability measurement, no noticeable change was observed in the XRD patterns and Raman spectra, revealing good structure stability (Supplementary Fig. 19b and c). In addition, the S 2*p* XPS spectra of ZrS_1-y_S_2-x_(15/100) after the repeated photoreaction show a weak peak located at ~168.7 eV (Supplementary Fig. 19d), suggesting a slight surface oxidation of ZrS_1-y_S_2-x_(15/100) after the photocatalytic measurement.

Based on the high activity of ZrS_1-y_S_2-x_(15/100) for H_2_O_2_ generation, we further utilized benzylamine to substitute the hole scavenger. The H_2_O_2_ evolution rate of ZrS_1-y_S_2-x_(15/100) was decreased to 78.1 ± 1.5 μmol h^−1^ with the same molar amount of benzylamine as benzyl alcohol, due to the slower oxidation kinetics of benzylamine than that of benzyl alcohol. Simultaneously, the benzylamine was oxidized and converted to benzonitrile at a rate of 32.0 ± 1.2 μmol h^−1^ with a high selectivity of >99% (entry 2 in Supplementary Table 2 and Fig. 4d), and no other by-products were detected by the Gas Chromatography-Mass Spectrometry measurements (Supplementary Fig. 20), consistent with the previous report^14,29^. Besides, the ZrS_1-y_S_2-x_(15/100) photocatalyst shows the rates for decomposition of H_2_O_2_ of 0.14 and 0.16 h^−1^ with the presence of benzyl alcohol and benzylamine, respectively, and the rates for formation of H_2_O_2_ of 125 and 113 μmol h^−1^ with the presence of benzyl alcohol and benzylamine, respectively (Supplementary Fig. 17b). Similar photocatalytic behaviors were also identified on both ZrSS_2-x_(15) and ZrS_3_ NBs, which produced the H_2_O_2_ at a rate of 58.5 ± 1.7 and 30.3 ± 1.3 μmol h^−1^ with the hole scavenger (entry 3 and 1 in Supplementary Table 2), respectively. As a comparison, ZrSS_2-x_(15) and ZrS_3_ show a decreased H_2_O_2_ evolution rate of 48.0 ± 1.2 and 18.1 ± 1.2 μmol h^−1^ with the use of benzylamine, and the corresponding benzonitrile generation rates are 20.7 ± 1.2 and 7.0 ± 1.0 μmol h^−1^, respectively (Fig. 4d). As a result, the comparison of photocatalytic performance among ZrS_1-y_S_2-x_(15/100), ZrSS_2-x_(15)_,_ and ZrS_3_ reveals the key role of S_2_^2−^ and S^2−^ vacancies on the O_2_ reduction and benzylamine oxidation.

**Fig. 5:**
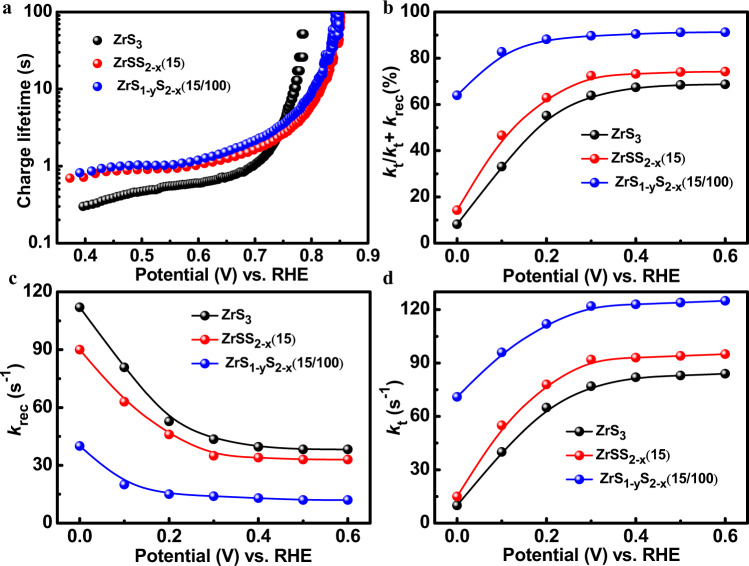
The charge carrier dynamics of the photocatalysts. **a** The charge carrier lifetime of ZrS_3_, ZrSS_2-x_(15), and ZrS_1-y_S_2-x_(15/100) NBs. **b** Ratio of *k*_t_/(*k*_t_ + *k*_rec_), rate constants **c** *k*_t_ and **d** *k*_rec_ of ZrS_3_, ZrSS_2-x_(15), and ZrS_1-y_S_2-x_(15/100) for benzylamine oxidation measured in 0.5 M Na_2_SO_4_ with 0.1 M benzylamine.

To provide a deep insight into the effect of defective structures in ZrS_3_ NBs on its photocatalytic performance, the transient open-circuit potential measurements were performed on ZrS_3_, ZrSS_2-x_(15), and ZrS_1-y_S_2-x_(15/100) NBs to reveal the lifetime of photo-induced charge carriers (Supplementary Fig. 21 and Supplementary Equation 4)^64^. After introducing S_2_^2−^ vacancies, the carrier lifetime of ZrSS_2-x_(15) was significantly increased to 0.69 s as compared to 0.3 s of ZrS_3_, while the ZrS_1-y_S_2-x_(15/100) exhibits a further enhanced lifetime of 0.82 s, as shown in Fig. 5a. The increased photocurrent for the defective ZrS_3_ also suggests the role of S_2_^2−^ and S^2−^ vacancies on improving the carrier lifetime and dynamics (Supplementary Fig. 22). In order to explore the underlying mechanism for the lifetime enhancement, the charge carrier dynamics of these samples were extracted through the Mott–Schottky method. According to the Mott–Schottky equation (Supplementary Equation 1), the electron concentrations of ZrS_3_, ZrSS_2-x_(15), and ZrS_1-y_S_2-x_(15/100) NBs were calculated to be 4.00 × 10^18^, 5.35 × 10^18,^ and 4.58 × 10^19^ cm^−3^, based on the estimated width of the depletion region (w_d_) under the illumination of 55, 46, and 17 nm, respectively (Supplementary Equation 2). The similar band bending between ZrS_3_ and ZrSS_2-x_(15) suggests that the significantly enhanced carrier lifetime in ZrSS_2-x_(15) is attributed to the role of S_2_^2−^ vacancies in reducing electron-hole recombination rather than band bending, in agreement with the previous theoretical calculation^41^. The significantly reduced w_d_ in ZrS_1-y_S_2-x_(15/100) indicates a large electric field strength on the surface of ZrS_1-y_S_2-x_(15/100), which can accelerate the extraction of photogenerated holes towards the surface and limit the internal band-to-band recombination. Moreover, the small w_d_ in ZrS_1-y_S_2-x_(15/100) results in a large conduction region for the free electrons compared to ZrS_3_ and ZrSS_2-x_(15), which is beneficial for the electron transport.

On the other hand, the reaction kinetics of benzylamine oxidation on the photocatalysts were also investigated by the intensity-modulated photocurrent spectroscopy (IMPS). The typical IMPS plots and the generalized reaction schematics are shown in Supplementary Fig. 23, and the details for the calculation of rate constant of charge transfer (*k*_t_) and surface recombination (*k*_rec_) are discussed in the supporting information. Since the *k*_t_/(*k*_t_ + *k*_rec_) can evaluate the efficiency of charge carrier transfer between the catalyst and reactant^43^, the clearly higher *k*_t_/(*k*_t_ + *k*_rec_) of ZrS_1-y_S_2-x_(15/100) than those of ZrSS_2-x_(15) and ZrS_3_ indicates that ZrS_1-y_S_2-x_(15/100) possesses higher efficiency for benzylamine oxidation (Fig. 5b). Furthermore, ZrSS_2-x_(15) presents a slightly higher *k*_t_/(*k*_t_ + *k*_rec_) compared to ZrS_3_, as derived from their similar behaviors of *k*_t_ and *k*_rec_ (Fig. 5c and d). The similar *k*_rec_ between ZrSS_2-x_(15) and ZrS_3_ is mainly ascribed to their similar surface band bending extracted from the Mott-Schottky results (Supplementary Fig. 7d). ZrS_1-y_S_2-x_(15/100) with the larger surface band bending thus shows a significantly decreased *k*_rec_. The slightly decreased *k*_rec_ of ZrSS_2-x_(15) compared to ZrS_3_ orginates from the suppression of surface charge recombination by S_2_^2−^ vacancies. The similar behavior of *k*_t_ for ZrSS_2-x_(15) and ZrS_3_ indicates that the introduction of S_2_^2−^ vacancies have a subtle effect on its catalytic capability for benzylamine oxidation. Furthermore, the large increase of *k*_t_ for ZrS_1-y_S_2-x_(15/100) compared to ZrSS_2-x_(15) and ZrS_3_ suggests that the S^2−^ vacancies can act as an additional photocatalytic layer for the benzylamine oxidation (Fig. 5d).

In summary, we have developed an efficient photocatalyst of ZrS_1-y_S_2-x_(15/100) NBs with S_2_^2−^ and S^2−^ vacancies for the integration of photocatalytic H_2_O_2_ generation with the selective oxidation of benzylamine to benzonitrile in water. More importantly, the unique S_2_^2−^ vacancies and S^2−^ vacancies can be controllably induced in the defective ZrS_3_ NBs by varying the annealing time and Li amount, which promise a prospective strategy for defect engineering. With the introduction of S_2_^2−^ vacancies, the charge carrier recombination is prominently suppressed, and the surface S^2−^ vacancies are revealed to improve the electron conduction, surface hole extraction, and kinetics of benzylamine oxidation. As a result, the photocatalyst of ZrS_1-y_S_2-x_(15/100) exhibits a high generation rate of 78.1 ± 1.5 and 32.0 ± 1.2 μmol h^−1^ for H_2_O_2_ and benzonitrile, respectively. Furthermore, ZrS_1-y_S_2-x_(15/100) NBs possesses a photoexcitation up to ~700 nm and delivers a high AQY of 11.4 and 10.8% under the incident light of 400 and 500 nm, respectively.

## Methods

### Preparation of ZrS_3_, ZrSS_2-x,_ and ZrS_1-y_S_2-x_ NBs

The ZrS_3_ NBs were synthesized through a typical chemical vapor transport process. 0.96 g S (99.5% purity, Alfa Aesar) and 0.91 g Zr (99.2% purity, Sigma-Aldrich) powders were mixed, and 5mg iodine (99.5% purity, Alfa Aesar) was added as a transport agent. The mixture was sealed in a quartz ampoule (Φ 6 mm × 200 mm) under the vacuum of 10^−3^ Pa, which was subsequently placed in the center of a two-zone furnace with a temperature gradient of ca. 15 K/cm from center to edge. The furnace was heated to 650 °C and last for 10 h to produce ZrS_3_ powder, which has a pure monoclinic crystal structure that is stable at this temperature. The obtained 1.87 g ZrS_3_ powder was then dispersed in isopropanol (≥99.5% purity, Alfa Aesar) at a concentration of 0.5 mg ml^−1^ followed by the sonication for 15 min. The dispersion was subsequently centrifuged for 10 min at 1006 xg to remove large aggregates. Finally, about 0.6 g ZrS_3_ NBs (32% yield) were obtained by the collection from the rest of the dispersion by further centrifugation for 10 min at 16099 xg.

Since hexagonal ZrS_2_ (ICCD PDF no. 11-0679) is usually obtained by vacuum annealing of monoclinic ZrS_3_ at elevated temperature (820 °C)^46^. This suggests that ZrS_3_ can be desulfurized into ZrS_2_ by the post-annealing at a higher temperature under vacuum. It is implied that such transformation can also be realized by the desulfuration of S_2_^2−^ ions, based on our previous results on TiS_3_ and crystal structure analysis between ZrS_2_ and ZrS_3_ in Fig. 1 and Supplementary Fig. 1^40^. As a result, the ZrSS_2-x_ NBs were prepared using the previously reported vacuum annealing method. Specifically, 0.6 g ZrS_3_ NBs were sealed in the quartz ampule (Φ 6 mm × 10 mm) again, which was then heated to 700 °C and last for different time (10, 15, and 20 mins) to fabricate ZrSS_2-x_ NBs. Besides, a certain amount of Li metal pieces (50, 100, and 150 mg) were added into 30 ml ethanediamine (≥98% purity, Sigma-Aldrich) for continuous magnetic stirring in an Ar-filled glovebox (O_2_, H_2_O < 0.1 ppm). After the Li was completely dissolved, 0.5 g ZrSS_2-x_ NBs were added into the solution, and the obtained solution was subsequently transferred into a 50 mL Teflon-lined autoclave and sealed immediately. Then, the Teflon-lined autoclave was taken out of the glovebox and kept in an oven at 120 °C for 24 h. After cooling down to room temperature, the mixture was first washed in 0.2 M HCl and then rinsed several times in deionized water and ethanol, where the ZrS_1-y_S_2-x_ NBs (yield > 96%) was finally obtained.

### Characterization of photocatalysts

UV-Vis-NIR spectrometer (Hitachi U4100), field emission SEM (FE-SEM, JEOL JSM6700F), TEM (FEI Titan 80-300, operated at 200 kV), XRD (Bruker D8 Advance), XPS (ESCALAB 250Xi) with Al K*a* X-ray as the excitation source, EPR (JEOL FA200), tapping-mode AFM (MPF-3D, Asylum Research, CA, USA), and Raman spectroscopy (Horiba Jobin Yvon Modular Raman Spectrometer) with 514 nm laser excitation were employed to characterize different properties of the defective ZrS_3_ NBs, e.g. atomic and energy band structure. In particular, the samples for the TEM measurements were suspended in ethanol and supported onto a holey carbon film on a Cu grid.

### Coupling photocatalytic H_2_O_2_ generation with selective benzylamine oxidation over ZrS_3_, ZrSS_2-x_, ZrS_1-y_S_2-x_ NBs

50 mg photocatalyst was dispersed in 30 ml H_2_O with 1 mmol benzylamine. After the sonication for a few seconds, the mixed solution was bubbled by oxygen for 30 s. Subsequently, the solution was sealed and irradiated under an AM 1.5G simulated sunlight of 100 mW cm^−2^ derived from a 300 W xenon lamp fitted with an AM 1.5 filter. At certain time intervals, the solution was filtrated by a 0.22 μm Millipore filter to remove the photocatalyst. The aqueous and organic phase products were then analyzed by the iodometry and gas chromatograph (GC) measurements, respectively.

The production of H_2_O_2_ was analyzed by the iodometry^12^. Typically, 50 μL 0.4 M potassium iodide (KI, ≥99% purity, Sigma-Aldrich) aqueous solution and 50 μL 0.1 M potassium hydrogen phthalate (≥99.5% purity, Sigma-Aldrich) aqueous solution were added to 2ml obtained aqueous phase product, which was kept for 0.5 h. The mixed solution was then detected by UV–vis spectroscopy on the basis of absorbance at 350 nm, from which the quantity of generated H_2_O_2_ was estimated. In addition, to analyze organic phase product from the benzylamine oxidation, the organic liquid was first extracted using ethyl acetate (≥99.9% purity, Sigma-Aldrich) and then detected by the GC characterization. Fihu

### Photocatalytic H_2_O_2_ generation with benzyl alcohol as hole sacrificial reagent

50 mg catalyst was dispersed in 30 ml H_2_O containing 1mmol benzyl alcohol. After sonicating for a few seconds, the mixed solution was bubbled by oxygen for few seconds. Subsequently, the solution was sealed and irradiated under an AM 1.5G simulated sunlight of 100 mW cm^−2^ derived from a 300 W xenon lamp fitted with an AM 1.5 filter. The amount of H_2_O_2_ was analyzed by the iodometry. For the action spectrum analysis, the reactions were performed at 298 K under monochromated light irradiation, with the *Φ*_AQY_ (AQY, apparent quantum yield) determined by the following Eq. (1):

1 $$\phi _{AQY}\left( \% \right) = \frac{{\left[ {{\mathrm{H}}_2{\mathrm{O}}_2\,{\mathrm{formed}}\,({\mathrm{mol}})} \right] \times 2}}{{{[\mathrm{photon}}\,{\mathrm{number}}\,{\mathrm{entered}}\,{\mathrm{into}}\,{\mathrm{the}}\,{\mathrm{reactor}}\,({\mathrm{mol}})]}} \times 100$$

### The stability test for ZrS_1-y_S_2-x_(15/100) NBs

The the ZrS_1-y_S_2-x_(15/100) NBs was recovered by centrifugation for 15 min at 16,099*g* and used for the reaction sequence, with water replaced every 3 h during photoirradiation.

### EPR trapping measurements

4 mg catalyst was suspended in 500 μL CH_3_OH containing 50 μL DMPO (Sigma-Aldrich for ESR-spectroscopy). After the sonication, the solution was irradiated by a 300 W xenon lamp with a 420 nm filter for 3 min. The resulted solution was subjected to the analysis by using a JEOL (FA200) ESR Spectrometer.

### Photoelectrochemical measurements

The photoelectrochemical measurements were performed in a three-electrode system with an electrochemical workstation (Zahner Zennium) under an AM 1.5G simulated sunlight of 100 mW cm^−2^ (150 W, Newport 94011A LCS-100). The samples on FTO substrates were firstly prepared by a typical electrophoretic deposition method. For details, 25 ml acetone solution containing 20 mg sample and 40 mg iodine was used as the electrophoresis solution. The experimental setup consists of two pieces of FTO that serve as the anodic and cathodic electrodes, respectively. The FTO substrates were immersed in the above solution in parallel with a distance of 1cm, which were kept in the solution for 5 min at a 10 V bias under the potentiostat control. After being calcined for 2 h in a vacuum oven at 100 °C, a uniform film was firmly coated on the FTO substrates. Samples on FTO substrates were directly used as the working electrode, with a Pt wire and an Ag/AgCl (KCl saturated) electrode as counter and reference electrodes respectively. All the samples were illuminated through the sample side (front-side illumination). The photoelectrochemical performance was recorded in 0.1 M Na_2_SO_4_ electrolyte with 0.1 mM benzylamine. Mott-Schottky plots were derived from impedance-potential tests conducted at a frequency of 1 kHz in dark. IMPS spectra were recorded by the Zahner Zennium C-IMPS system.

## Supplementary information

Supplementary Information

Peer Review File

## Data Availability

All data supporting the findings in the article as well as the Supplementary Information files are available from the corresponding authors on reasonable request.
