# Supplementary material for: Efficient photocatalytic hydrogen peroxide generation coupled with selective benzylamine oxidation over defective ZrS3 nanobelts
Source: Nat Commun. 2021 Apr 1;12:2039. doi: 10.1038/s41467-021-22394-8 (PMC8016833; doi:10.1038/s41467-021-22394-8)
Supplement: Supplementary file 1 — Supplementary Information [file 41467_2021_22394_MOESM1_ESM.pdf]

SUPPLEMENTARY INFORMATION

Efficient Photocatalytic Hydrogen Peroxide Generation Coupled with Selective Benzylamine Oxidation over Defective ZrS_3 Nanobelts

Tian et al.

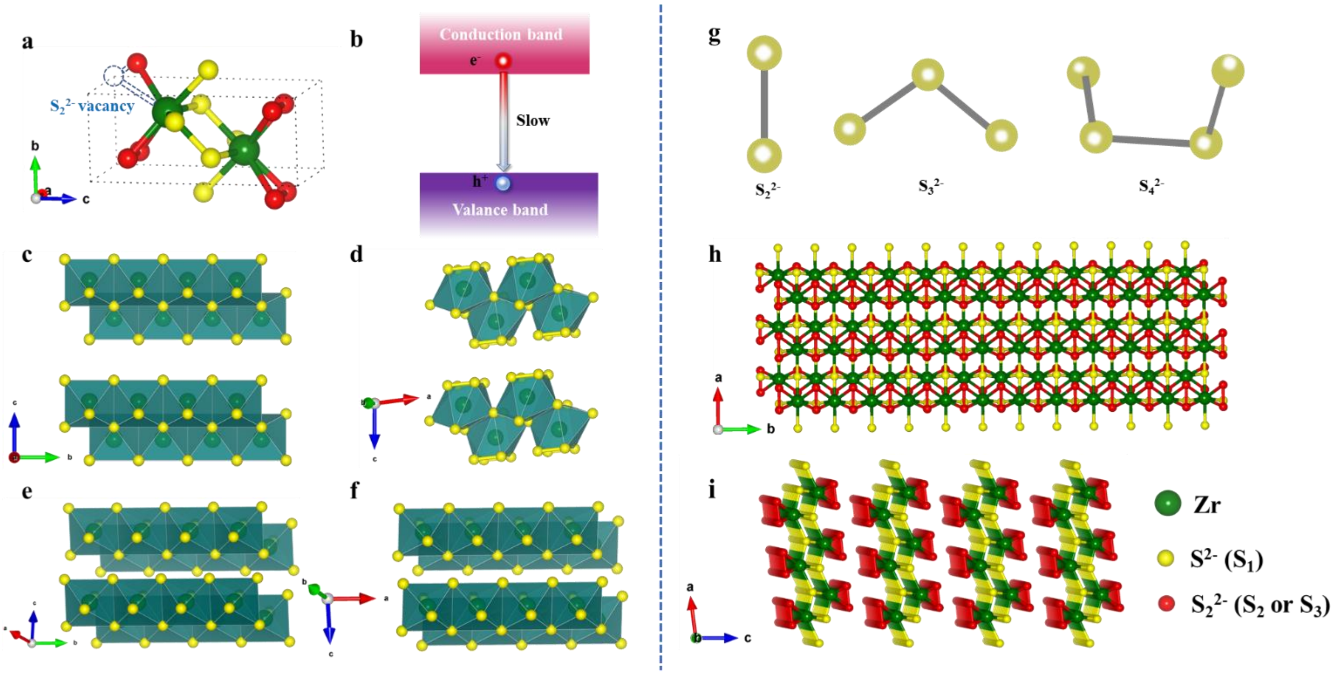

Supplementary Figure 1. The crystal structure of ZrS_3 and ZrS_2. (a) ZrS_3 unit cell with a S_2^{2-} vacancy and (b) slow electron-hole recombination of $ZrSS_{2-x}$. (c-f) Comparison of the crystal structures between monoclinic ZrS_3 (ICCD PDF no. 30-1498) and hexagonal ZrS_2 (ICCD PDF no. 11-0679). (c, d) Crystal structure of ZrS_3 with [100] and [010] views, respectively. (e, f) Crystal structure of ZrS_2 with [100] and [010] views, respectively. (g) Structure of S_x^{2-} ($x \geq 2$) ions. It is very difficult for S_x^{2-} ($x \geq 2$) to interact with other metals to form chemical bonds. (h, i) Crystal structure of ZrS_3 NB with different views.

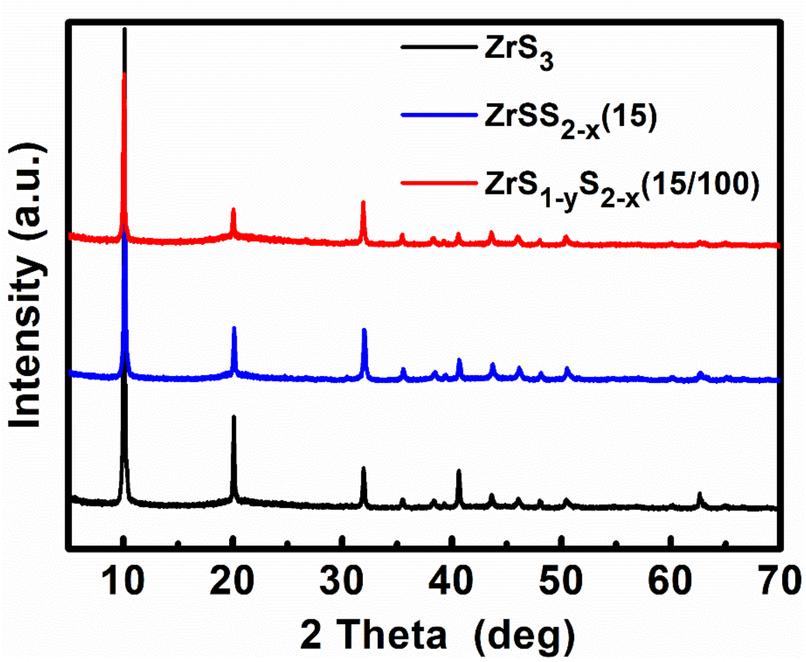

Supplementary Figure 2. XRD spectra of ZrS_3, $ZrSS_{2-x}$(15), and $ZrS_{1-y}S_{2-x}$(15/100) NBs. The x-ray diffraction (XRD) spectrum confirms the formation of ZrS_3 in the monoclinic phase (ICCD PDF no. 30-1498), and the vacuum annealing and further Li treatment did not induce any phase transition in $ZrSS_{2-x}$(15) and $ZrS_{1-y}S_{2-x}$(15/100) NBs.

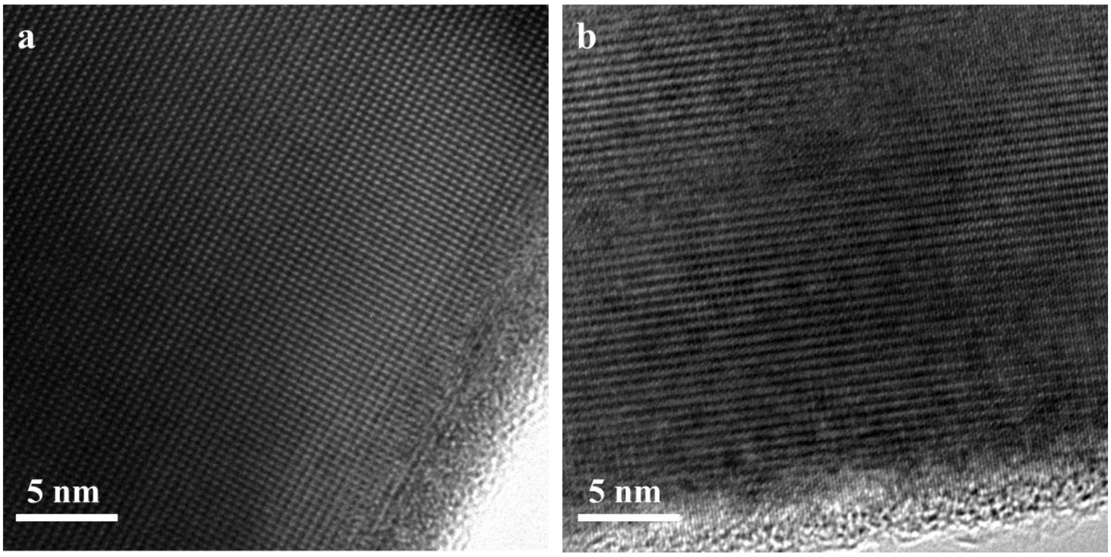

Supplementary Figure 3. HRTEM images of (a) ZrS_3 and (b) $ZrS_{1-y}S_{2-x}$(15/100) NBs.

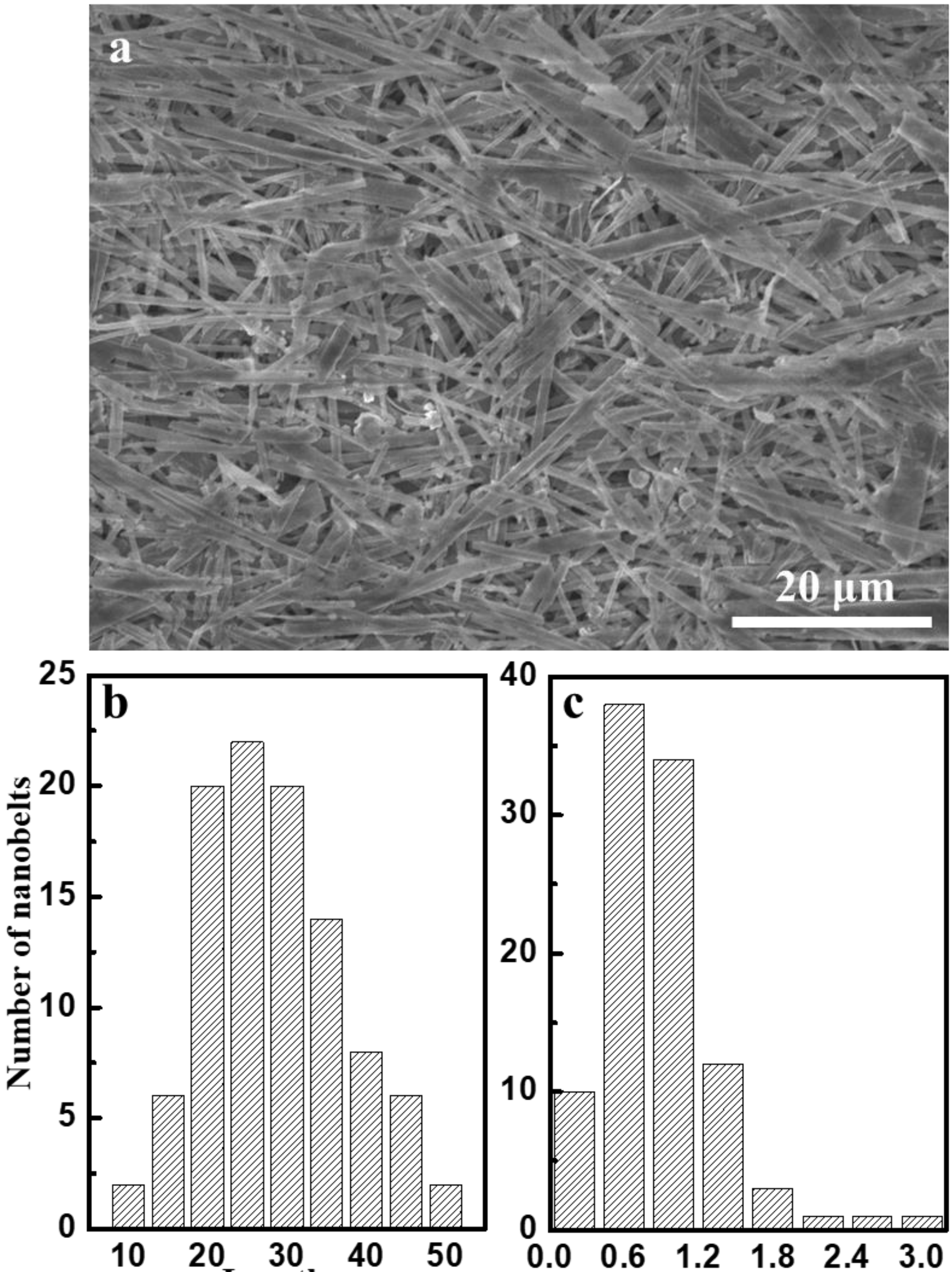

Supplementary Figure 4. Statistical length distribution of ZrS_3 NBs. (a) SEM micrograph of ZrS_3 NBs and the corresponding (b) length and (c) width distribution. To have a clear view of the length of ZrS_3 NBs, the sample for SEM measurements was prepared by a suction filtration method, and the pore diameter of the used filter membrane is 0.22 μm.

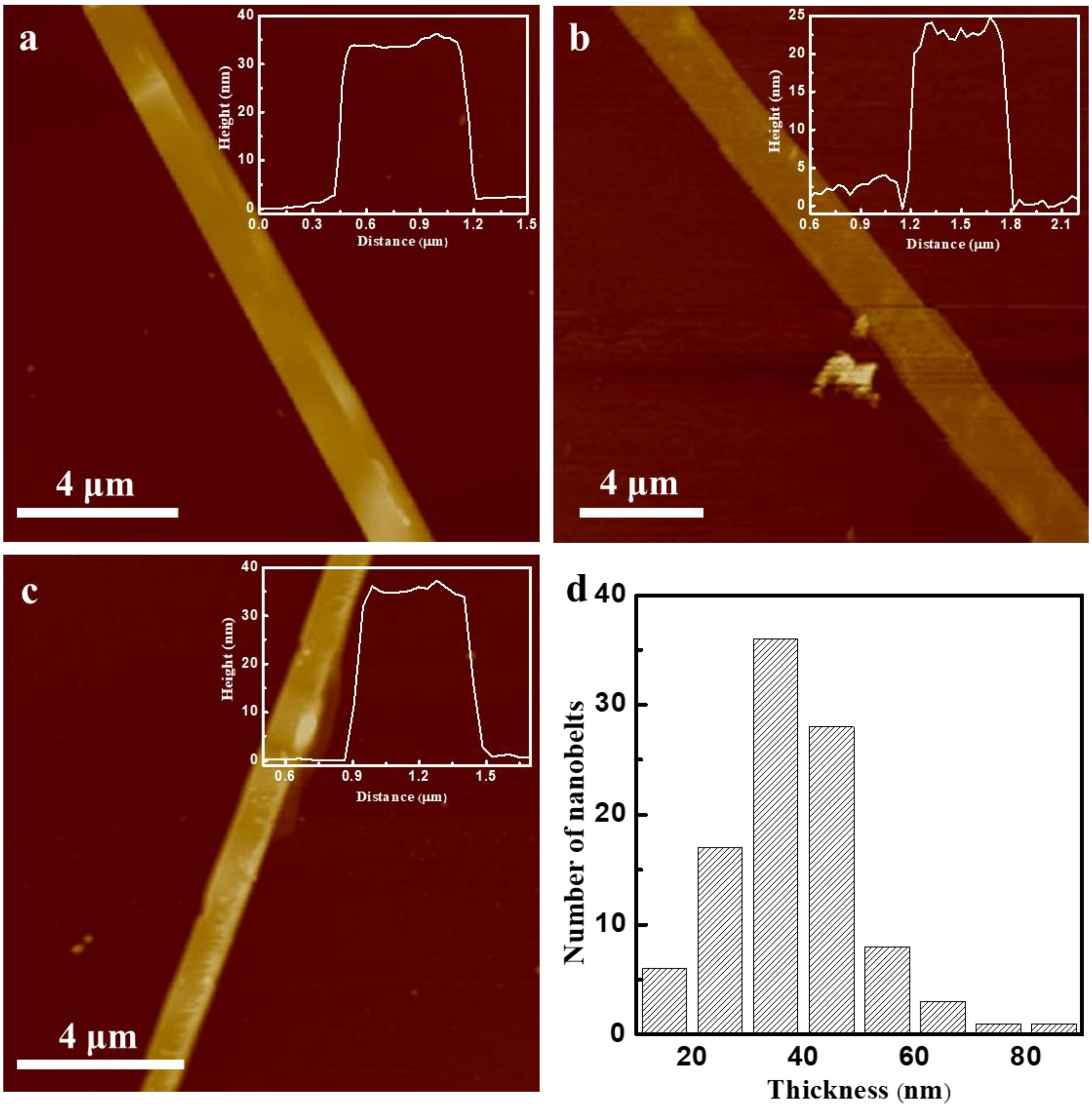

Supplementary Figure 5. Statistical width and thickness distribution of ZrS_3 NBs. (a)-(c) Representative AFM images of ZrS_3 NBs. Inset: the corresponding height profiles. (d) Thickness distribution of ZrS_3 NBs extracted from AFM measurements by imaging 100 ZrS_3 NBs.

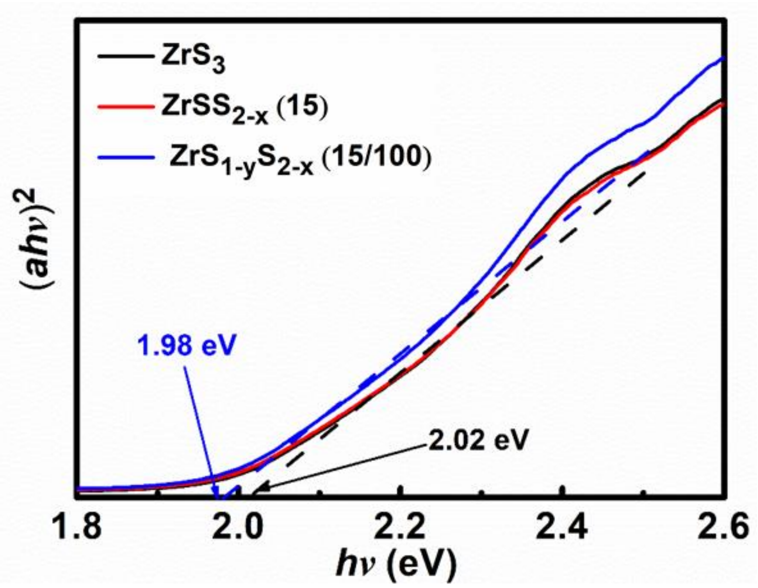

Supplementary Figure 6. Tauc plots of the ZrS_3, $ZrSS_{2-x}$(15), and $ZrS_{1-y}S_{2-x}$(15/100) NBs. Since ZrS_3 is an indirect bandgap semiconductor,[1] the bandgaps of ZrS_3, $ZrSS_{2-x}$(15), and $ZrS_{1-y}S_{2-x}$(15/100) NBs are estimated to be 2.02, 2.02, and 1.98 eV respectively, based on their absorption spectra (**Figure 2e**).

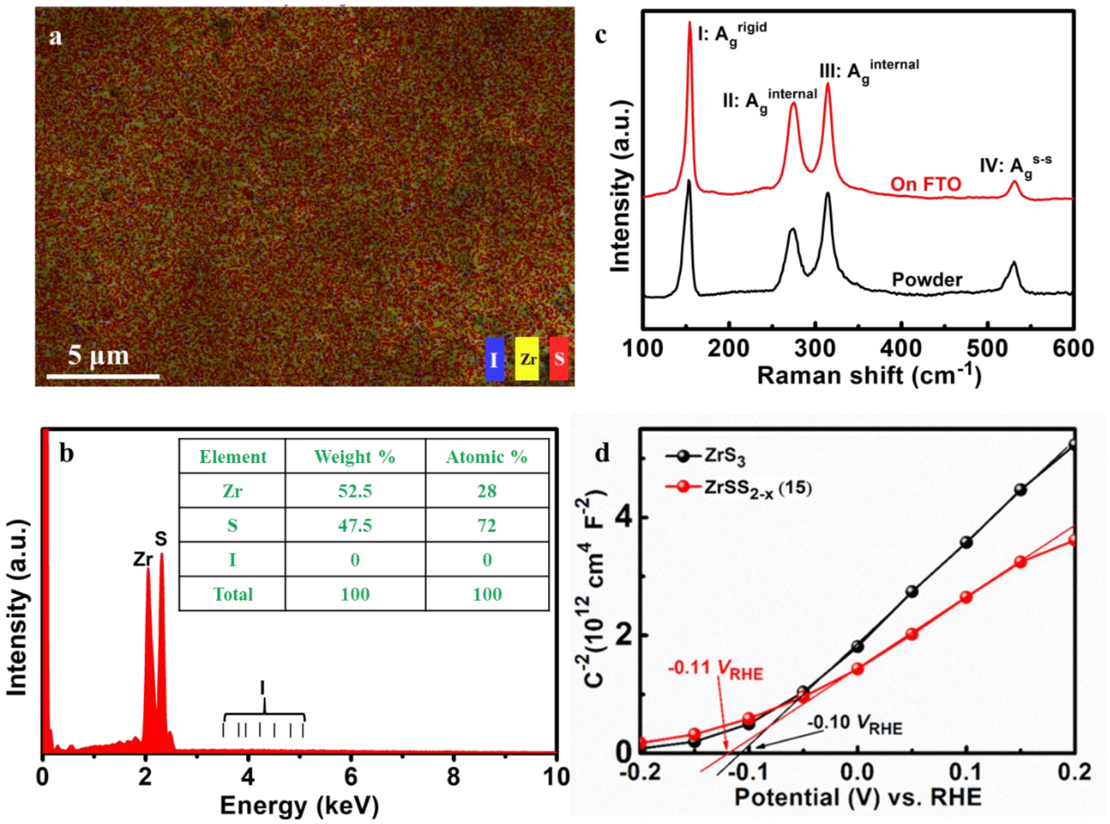

Supplementary Figure 7. Characterizations of the samples deposited on the FTO substrate. (a) SEM-EDS mapping image of the $ZrS_{1-y}S_{2-x}$(15/100) NBs deposited on the FTO substrate, and (b) the corresponding EDS spectrum. (c) Raman spectra of the $ZrS_{1-y}S_{2-x}$(15/100) NBs before and after the deposition on the FTO substrate. (d) Mott–Schottky plots of ZrS_3 and $ZrSS_{2-x}$(15) NBs magnified from **Figure 2f** to have a view of their E_{fb}s. Mott−Schottky plots recorded at 1000 Hz with a 5 mV amplitude in the 0.5 M Na_2SO_4 solution with 0.1 M benzylamine (without illumination).

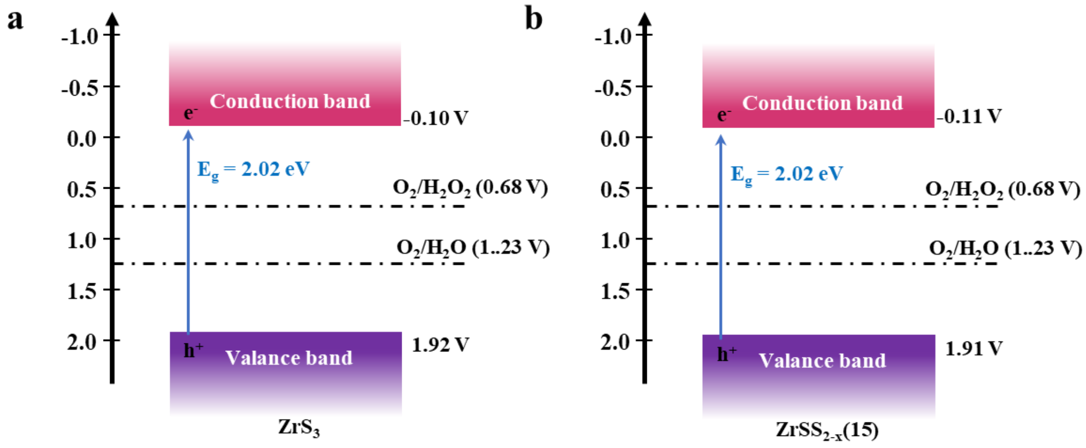

Supplementary Figure 8. Schematic diagrams of band structures for (a) ZrS_3 and (b) $ZrSS_{2-x}$(15).

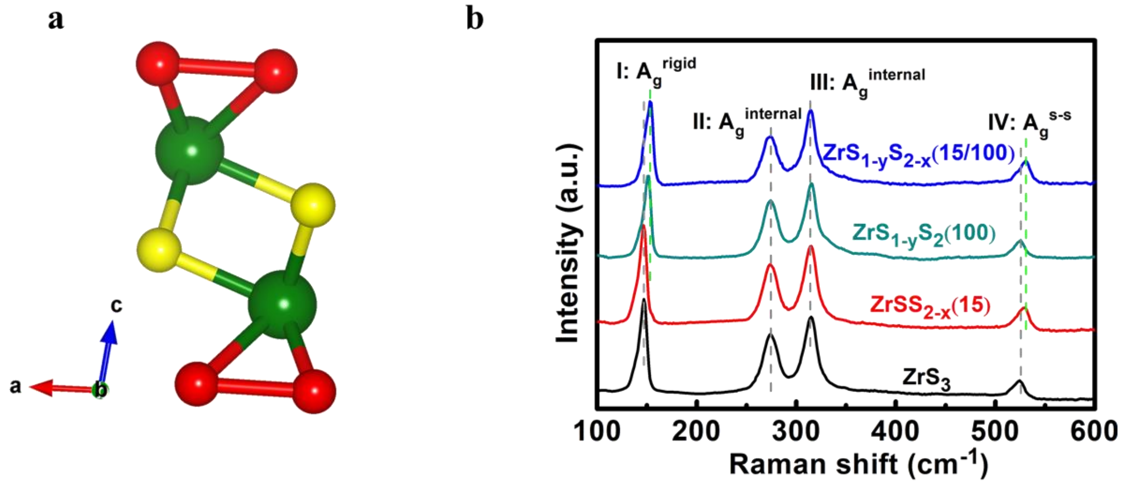

Supplementary Figure 9. Raman properties of the defective ZrS_3 NBs. (a) Schematic showing the Raman active mode of I: A_g^{rigid}. A_g^{rigid} is related to the vibration of quasi-one-dimensional chains in the direction of the c axis, which keeps the Zr–S bond length fixed within each chain. (b) Raman spectra of the ZrS_3, $ZrSS_{2-x}$(15), $ZrS_{1-y}S_2$(100), and $ZrS_{1-y}S_{2-x}$(15/100) NBs.

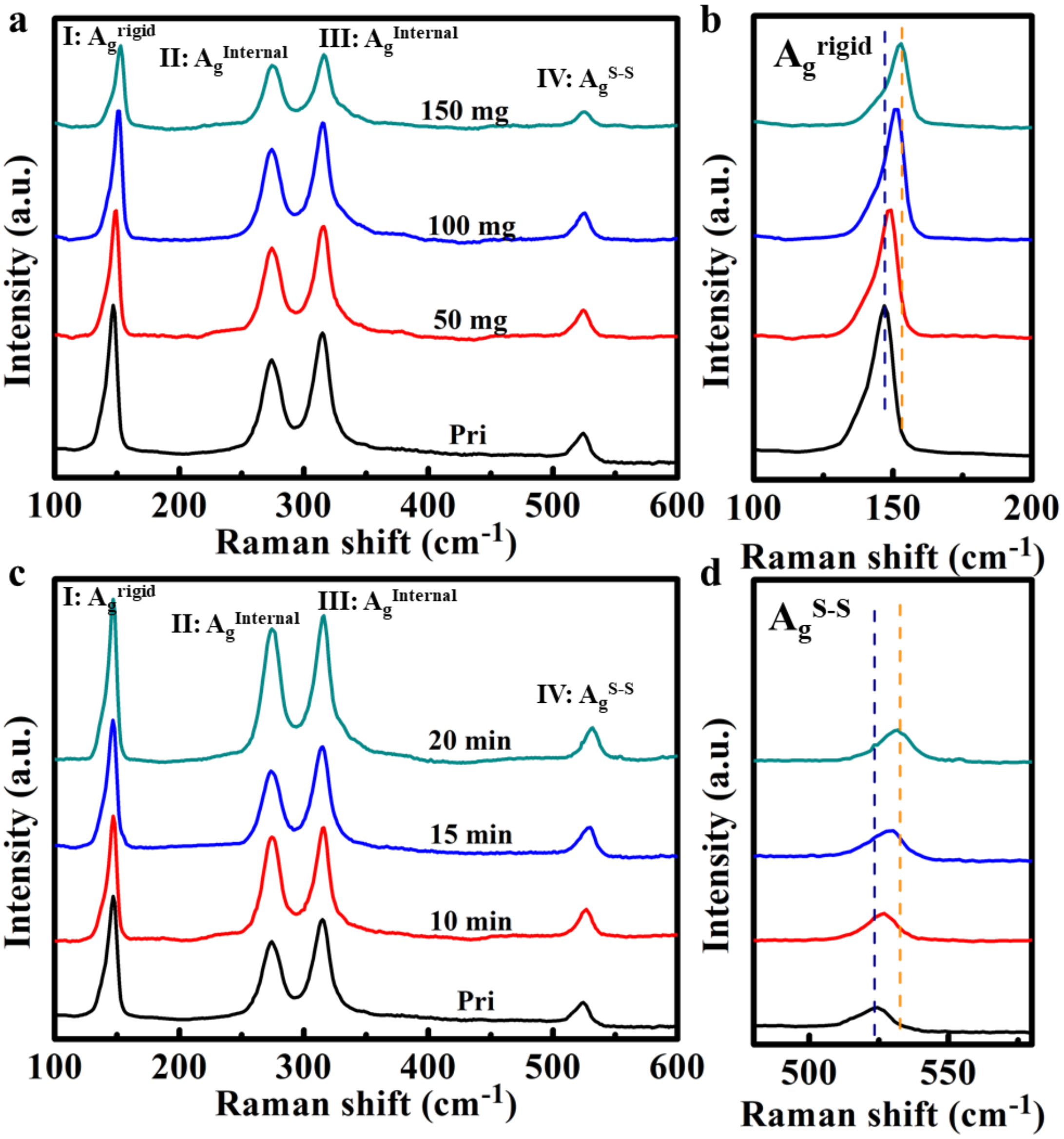

Supplementary Figure 10. Representative Raman spectra of the defective ZrS_3 NBs. (a) Raman spectra of the ZrS_3 NBs treated by the Li-treatment with different Li amount. (b) The enlarged A_g^{rigid} model from (a). (c) Raman spectra of the ZrS_3 NBs treated by the vacuum annealing for different time. (d) The enlarged $A_g^{s\text{–}s}$ model from (c).

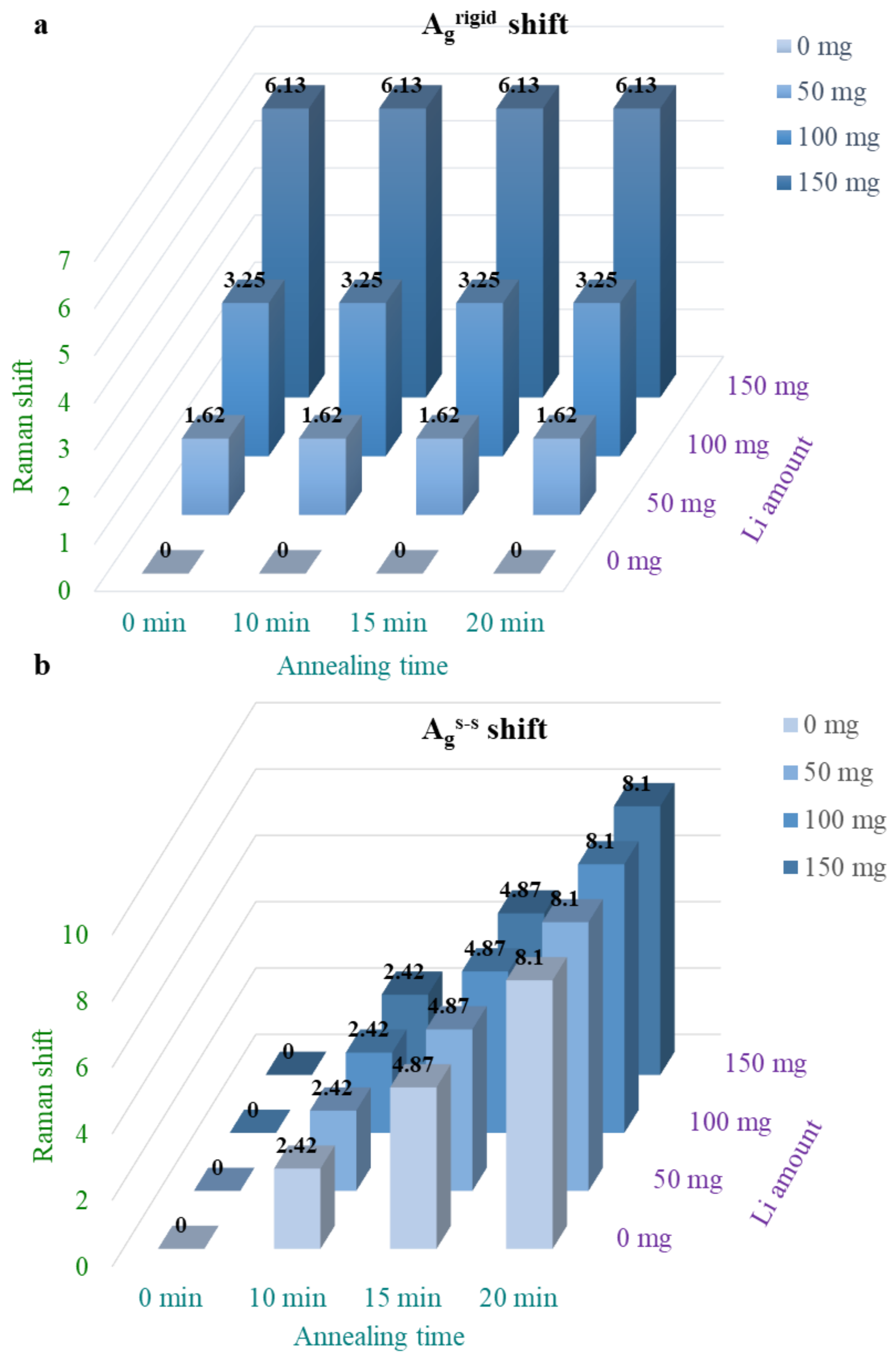

Supplementary Figure 11. 3D histograms of the (a) A_g^{rigid} shift and (b) $A_g^{s\text{–}s}$ shift for different samples.

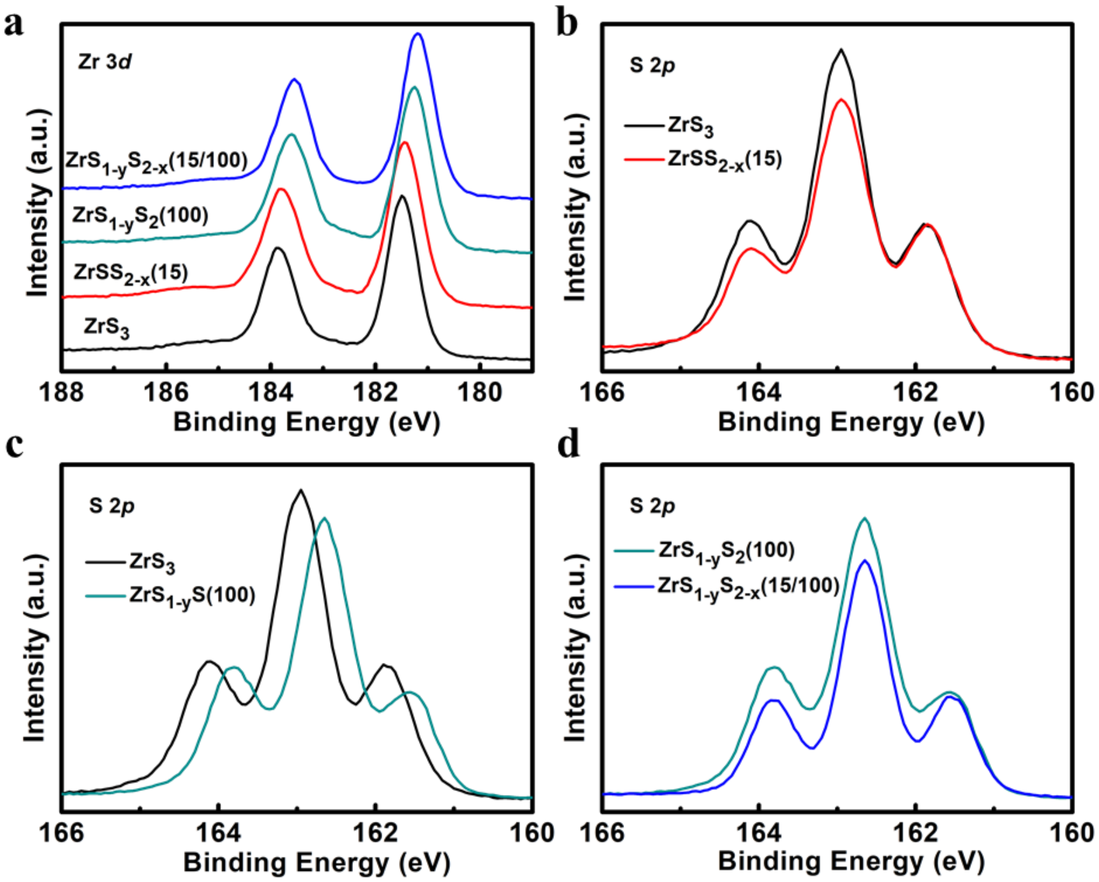

Supplementary Figure 12. XPS results of the defective ZrS_3 NBs. (a) Zr 3d XPS spectra of ZrS_3, $ZrSS_{2-x}$(15), $ZrS_{1-y}S_2$(100), and $ZrS_{1-y}S_{2-x}$(15/100). (b) S 2p XPS spectra of ZrS_3 and $ZrSS_{2-x}$(15). (c) S 2p XPS spectra of ZrS_3 and $ZrS_{1-y}S_2$(100). (d) S 2p XPS spectra of $ZrS_{1-y}S_2$(100) and $ZrS_{1-y}S_{2-x}$(15/100).

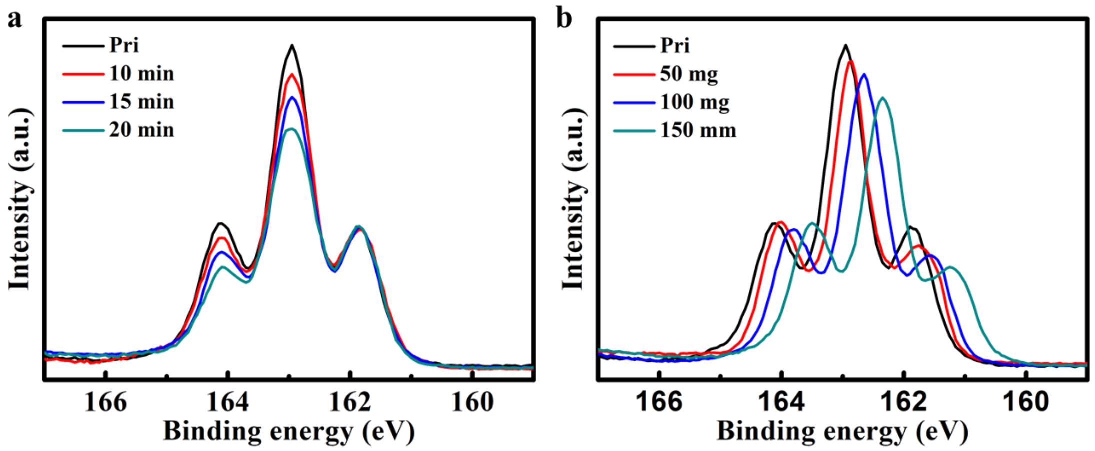

Supplementary Figure 13. Representative S 2p XPS spectra of the defective ZrS_3 NBs. (a) S 2p XPS spectra of the ZrS_3 NBs treated by vacuum annealing for different time. (b) S 2p XPS spectra of the ZrS_3 NBs treated with different Li amount.

Supplementary Table 1. The summary of x and y values of $ZrS_{1-y}S_{2-x}$ NBs by different treatments, as extracted from the XPS measurements.

Annealing time \ Li content	0	50 mg	100 mg	150 mg
0 min	x = 0<br>y = 0	x = 0<br>y = 0.05	x = 0<br>y = 0.14	x = 0<br>y = 0.18
10 min	x = 0.21<br>y = 0	x = 0.21<br>y = 0.06	x = 0.20<br>y = 0.15	x = 0.19<br>y = 0.18
15 min	x = 0.37<br>y = 0	x = 0.37<br>y = 0.05	x = 0.36<br>y = 0.13	x = 0.36<br>y = 0.19
20 min	x = 0.49<br>y = 0	x = 0.48<br>y = 0.06	x = 0.50<br>y = 0.15	x = 0.49<br>y = 0.20

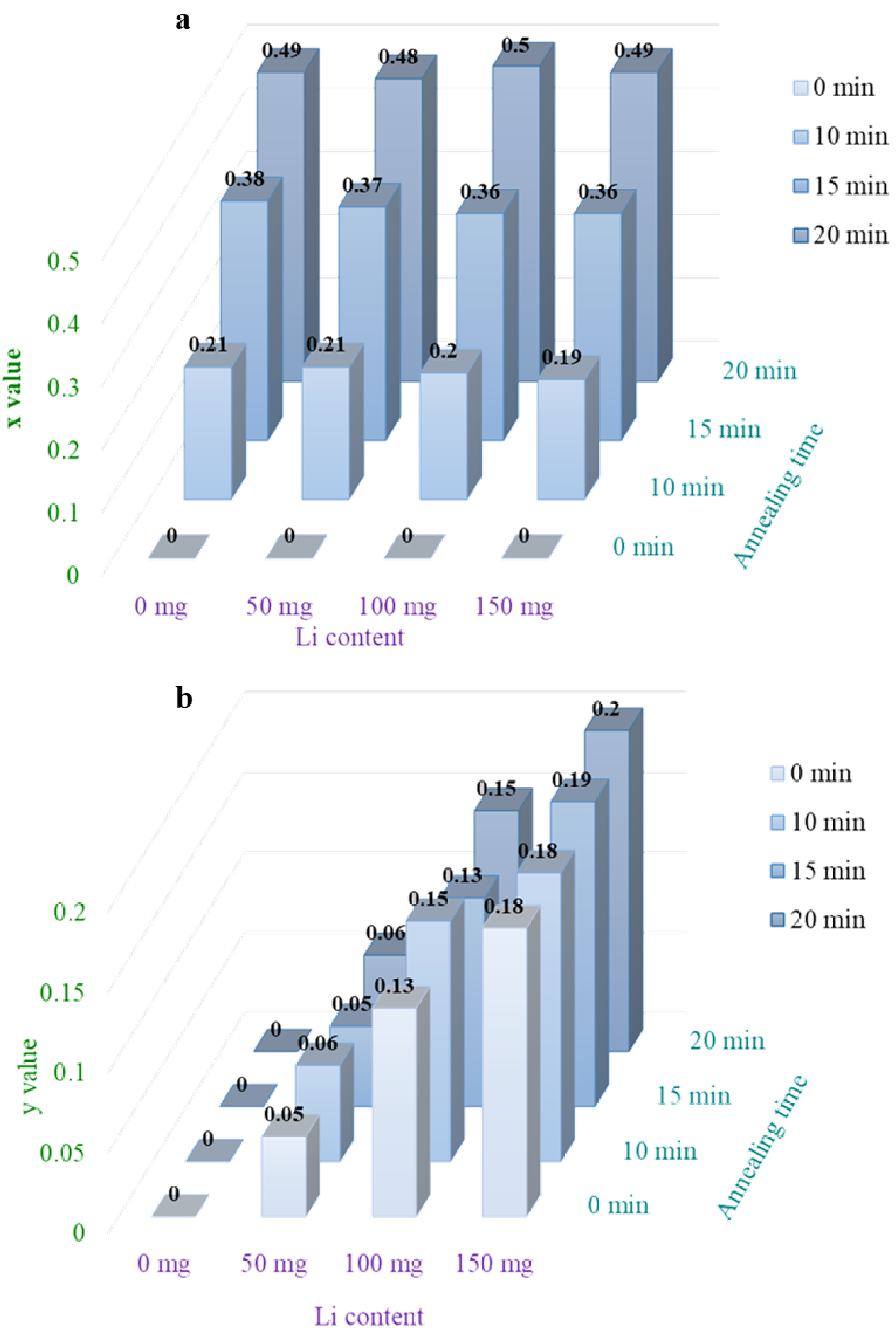

Supplementary Figure 14. 3D histograms of the extracted (a) x and (b) y values for different samples from XPS measurements.

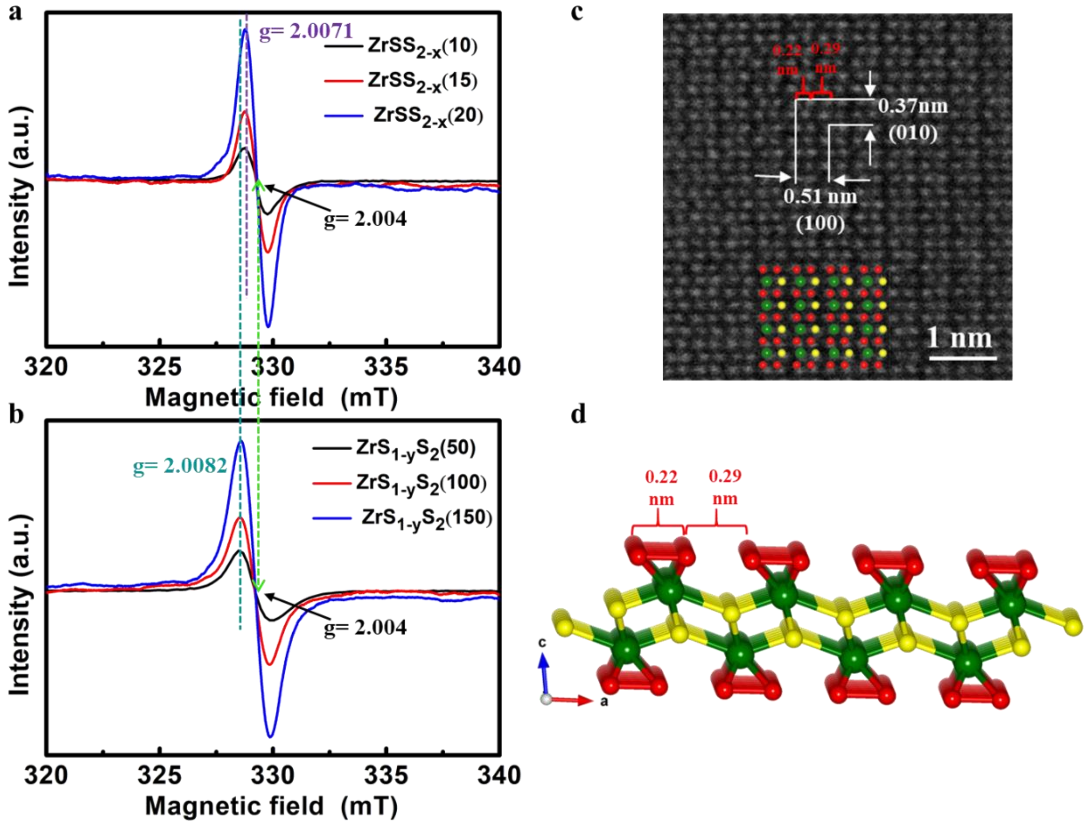

Supplementary Figure 15. Representative EPR spectra of the defective ZrS_3 NBs and the crystal structure of ZrS_3. (a) EPR spectra of the ZrS_3 NBs treated by vacuum annealing for different time. (b) EPR spectra of the ZrS_3 NBs treated with different Li amount. (c) HAADF-STEM images of ZrS_3. (d) Crystal structure of the ZrS_3 NB from the [010] view.

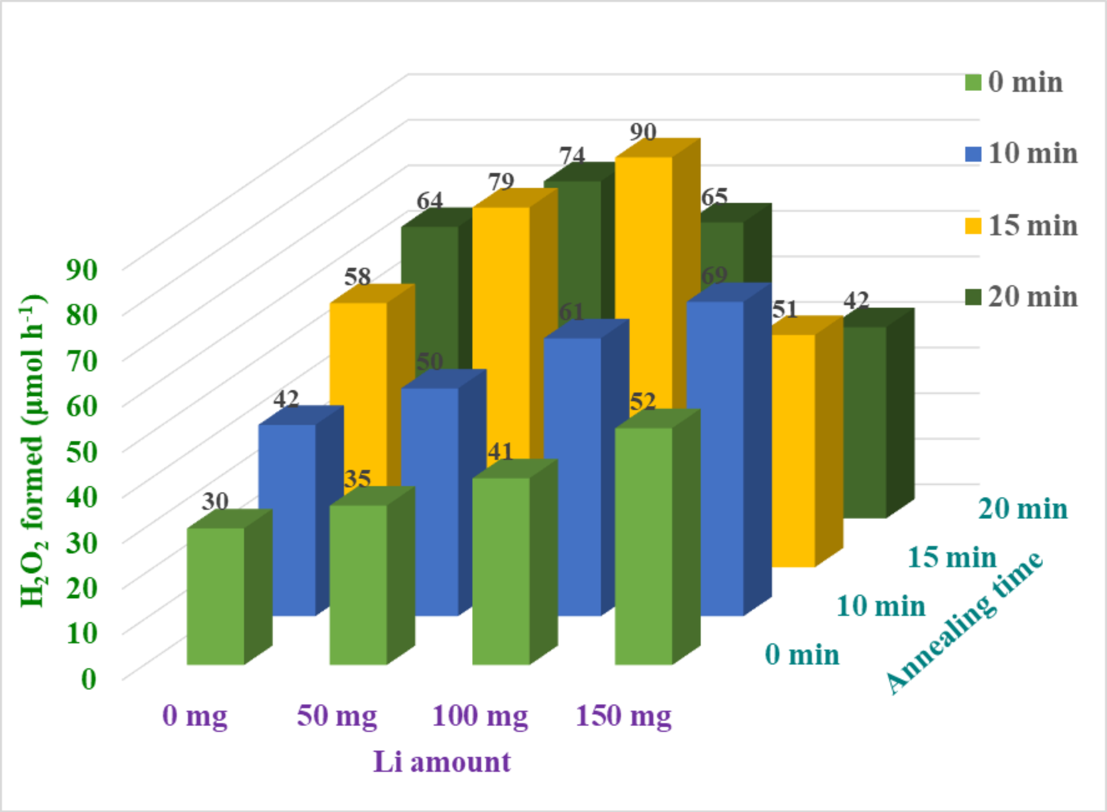

Supplementary Figure 16. The activity of photocatalytic H_2O_2 generation for different defective ZrS_3 NBs under AM1.5G simulated sunlight irradiation. Conditions: 30 ml aqueous solution with 1 mmol benzyl alcohol, 50 mg photocatalysts.

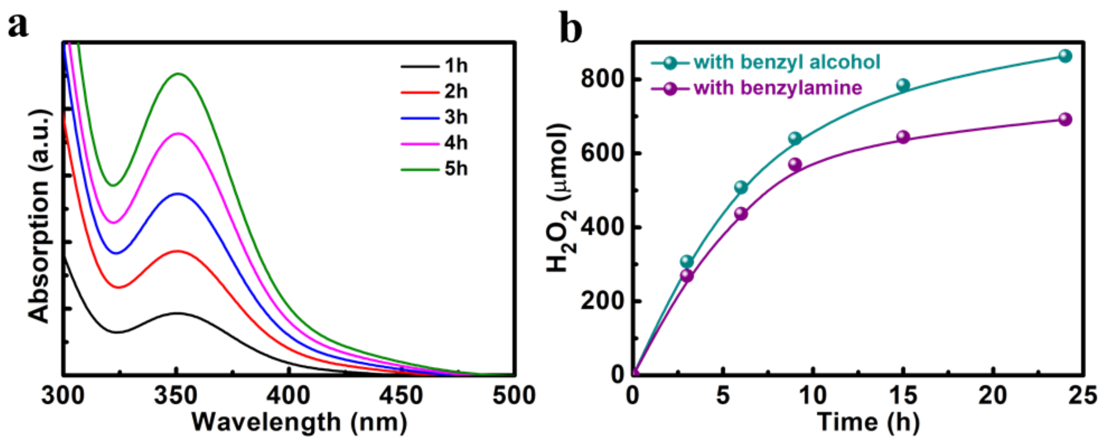

Supplementary Figure 17. Time-dependent formation of H_2O_2 over the $ZrS_{1-y}S_{2-x}$(15/100) photocatalyst. (a) UV-vis absorption spectrum changes of H_2O_2 generation of $ZrS_{1-y}S_{2-x}$(15/100) under AM1.5G simulated sunlight irradiation. Conditions: 30 ml aqueous solution with 1 mmol benzylamine, 50 mg photocatalysts. (b) Time-dependent change in H_2O_2 concentration during photoreaction for the $ZrS_{1-y}S_{2-x}$(15/100) photocatalyst with the presence of benzyl alcohol and benzylamine, respectively.

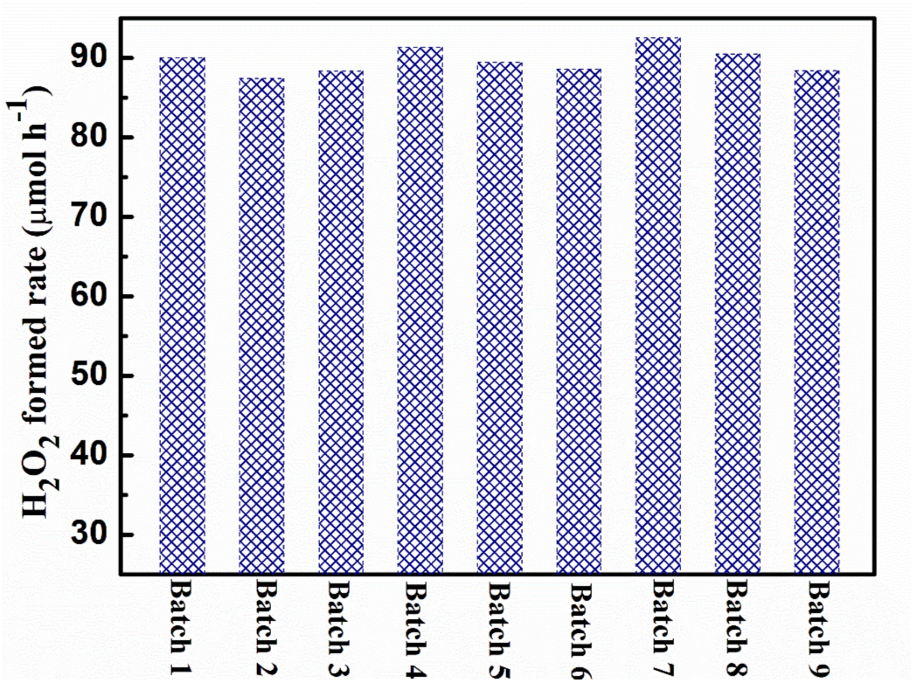

Supplementary Figure 18. Reproducibility of H_2O_2 generation for $ZrS_{1-y}S_{2-x}$(15/100) NBs from 9 batches. Conditions: AM1.5G simulated sunlight irradiation, 30 ml aqueous solution with 1 mmol benzyl alcohol, 50 mg photocatalysts, 5 h duration for the reaction. Different batches represent the samples synthesized at different timing. The average of the H_2O_2 generation rate was determined to be 89.6 μmol h^{-1} with the standard deviation of 1.54 μmol h^{-1}.

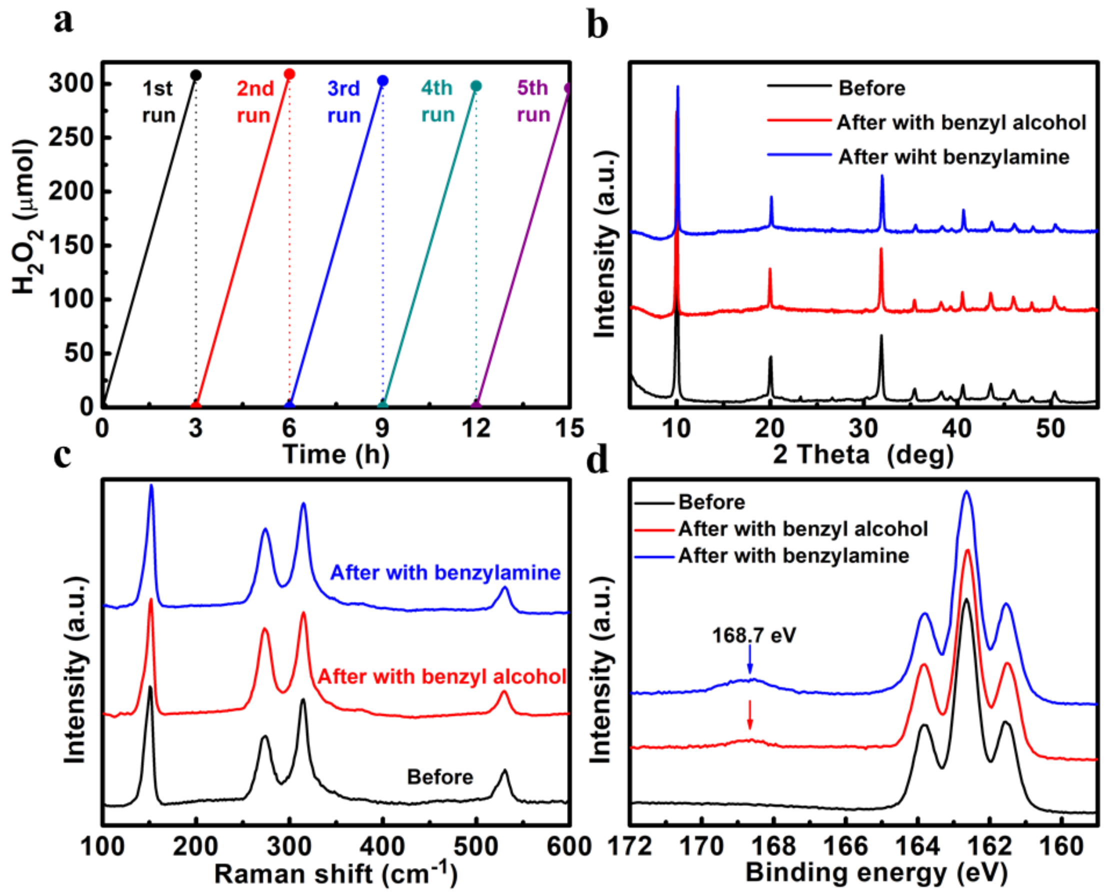

Supplementary Figure 19. The stability of the $ZrS_{1-y}S_{2-x}$(15/100) photocatalyst. (a) Results of H_2O_2 generation for a repeated photoreaction sequence with $ZrS_{1-y}S_{2-x}$(15/100) under AM1.5G simulated sunlight irradiation. Conditions: 30 ml aqueous solution with 1 mmol benzyl alcohol, 50 mg photocatalysts. The comparison of (b) XRD patterns, (c) Raman spectra, and (d) S 2p XPS spectra of the $ZrS_{1-y}S_{2-x}$(15/100) NBs before and after the 15 h photocatalytic test.

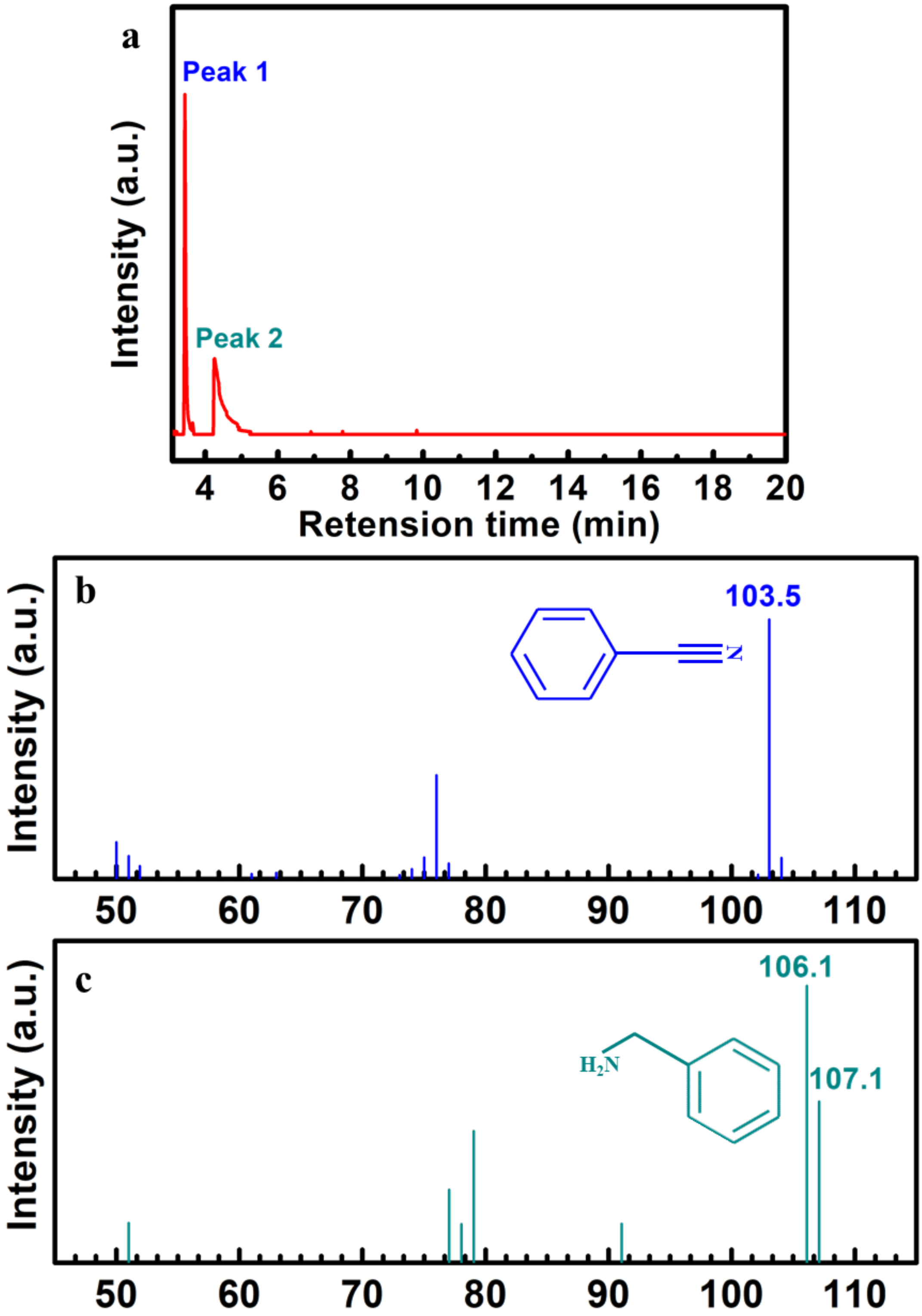

Supplementary Figure 20. Gas Chromatography-Mass Spectrometry (GC-MS) analysis for $ZrS_{1-y}S_{2-x}$(15/100). (a) GC spectrum of the extracted ethyl acetate solution. Corresponding MS spectra of (b) peak 1 and (c) peak 2. Conditions: 30 ml aqueous solution with 1 mmol benzylamine, 50 mg photocatalysts, 5 h reaction time, AM1.5G simulated sunlight irradiation.

Supplementary Table 2. The activity of photocatalytic H_2O_2 generation and/or benzylamine oxidative coupling reaction.[a] Errors are the standard error of the mean for 9 independent samples.

Entry	Catalyst	BN rate (μmol h^{-1})	Sel (%)[c]	H_2O_2 rate (μmol h^{-1})
1[b]	ZrS_3	-	-	30.3 ± 1.3
2	ZrS_3	7.0 ± 1.0	>99%	18.1 ± 1.2
3[b]	$ZrSS_{2-x}$(15)	-	-	58.5 ± 1.7
4	$ZrSS_{2-x}$(15)	20.7 ± 1.2	>99%	48.0 ± 1.2
5[b]	$ZrS_{1-y}S_{2-x}$(15/100)	-	-	89.6 ± 1.5
6	$ZrS_{1-y}S_{2-x}$(15/100)	32.0 ± 1.2	>99%	78.1 ± 1.5

[a] Reaction conditions: 30 ml aqueous solution with 1 mmol benzylamine, 50 mg catalysts, 1 atm O_2, AM1.5G simulated sunlight (1 sun) irradiation. [b] 1 mmol benzyl alcohol instead of benzylamine. [c] Determined by GC analysis.

Supplementary Table 3. A detailed comparison of photocatalytic H_2O_2 production by recently reported state-of-the-art oxide-based photocatalysts.

Material	Reaction conditions	Irradiation conditions	H_2O_2 yield	AQY	Ref
$ZrS_{1-y}S_{2-x}$(15/100)	O_2; 1.67 g/L (catalyst); water + benzyl alcohol	AM1.5G simulated sunlight, 100 mW cm^{-2}	450 μmol (5h)	11.4 and 10.8 % at 400 and 500 nm	This work
$ZrS_{1-y}S_{2-x}$(15/100)	O_2; 1.67 g/L; water + benzylamine	AM1.5G simulated sunlight, 100 mW cm^{-2}	390 μmol (5h)	-	This work
Resins	O_2; 1.67 g/L; water	Xe-lamp, 420-700 nm, 14 mW cm^{-2}	99 μmol (24h)	8 % at 420 nm	2
Oxygen-enriched g-C_3N_4	O_2; 1.67 g/L; water + 2-propanol	Xe-lamp, λ≥420nm, 35.2 mW cm^{-2}	730 μmol (5h)	28.5 and 10.2 % at 365 and 420 nm	3
g-C_3N_4/PDI/$rGO_{0.05}$	O_2; 1.67 g/L; water	Xe-lamp, 420-500 nm, 4.3 mW cm^{-2}	29 μmol (24h)	6.1 % at 420 nm	4
(K, P, O)-g-C_3N_4	O_2; 0.5 g/L; water + ethanol	Xe-lamp, λ≥420nm, 726.8 mW cm^{-2}	1.7 mm (7 h)	26.2 and 8 % at 320 and 420 nm	5
g-C_3N_4/PDI51	O_2; 1.67g/L; water + 2-propanol	Xe-lamp, 420−500 nm, 2.69 mW cm^{-2}	210 μmol (6h)	-	6
CdS-graphene oxide	O_2; 0.5 g/L; water + methanol	λ = 420nm, 23 mW cm^{-2}	95 μM (1h)	-	7
m-WO_3/FTO-Co^{II}(Ch)/CP	O_2; seawater	AM1.5G simulated sunlight, 100 mW cm^{-2}	48 mM (24 h)	-	8
MIL-125-NH_2	O_2; 0.71 g/L; benzyl alcohol/water	λ≥420nm	2.4 mM (3 h)	-	9
rGO/$Cd_3(TMT)_2$	O_2; 4 g/L; water + methanol	λ≥420nm	7 mM (24 h)	6.8 % at 450 nm	10
Au/MoS_2	O_2; 1 g/L; water + methanol	Real sunlight irradiation	791.72 μM (6h)	-	11

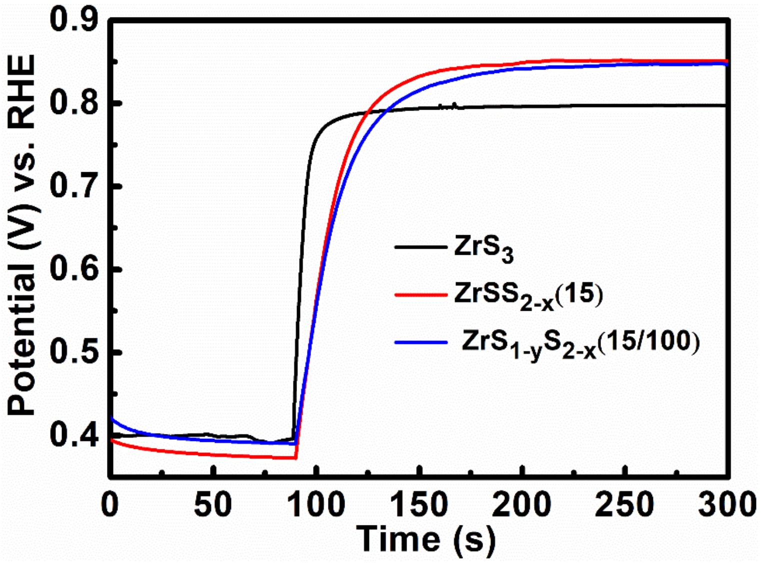

Supplementary Figure 21. Open-circuit potentials of the ZrS_3, $ZrSS_{2-x}$(15), and $ZrS_{1-y}S_{2-x}$(15/100) NBs as a function of time in 0.5 M Na_2SO_4 with 0.1 M benzylamine.

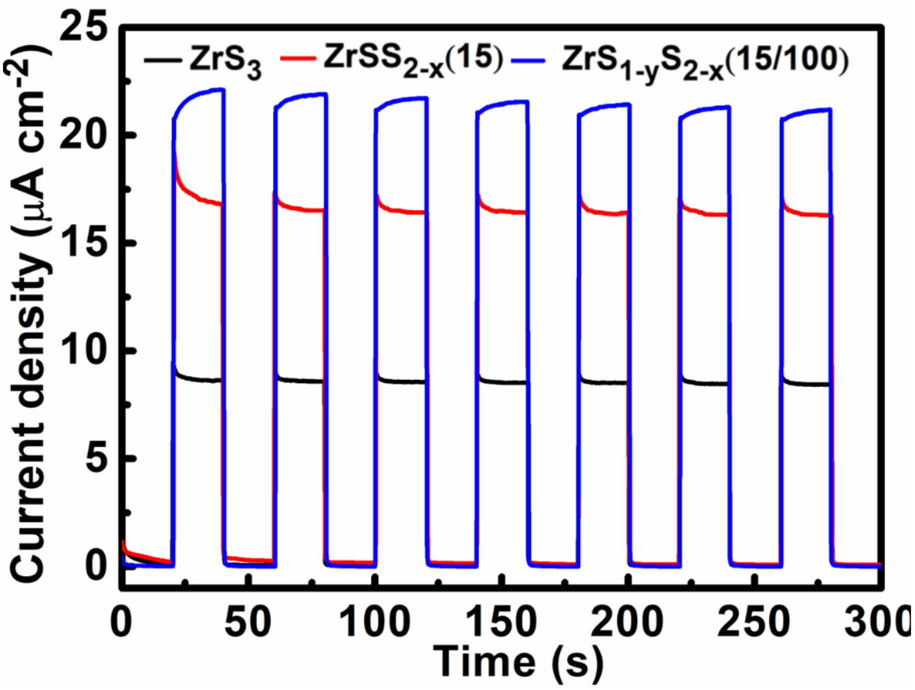

Supplementary Figure 22. Periodic on/off photocurrent response measured in 0.5 M Na_2SO_4 with 0.1 M benzylamine at 0.4 V_{RHE} for the ZrS_3, $ZrSS_{2-x}$(15), and $ZrS_{1-y}S_{2-x}$(15/100) NBs. The enhanced photocurrent densities of the $ZrSS_{2-x}$(15), and $ZrS_{1-y}S_{2-x}$(15/100) NBs indicate that both S_2^{2-} and S^{2-} vacancies play a key role in improving the carrier lifetime and dynamics.

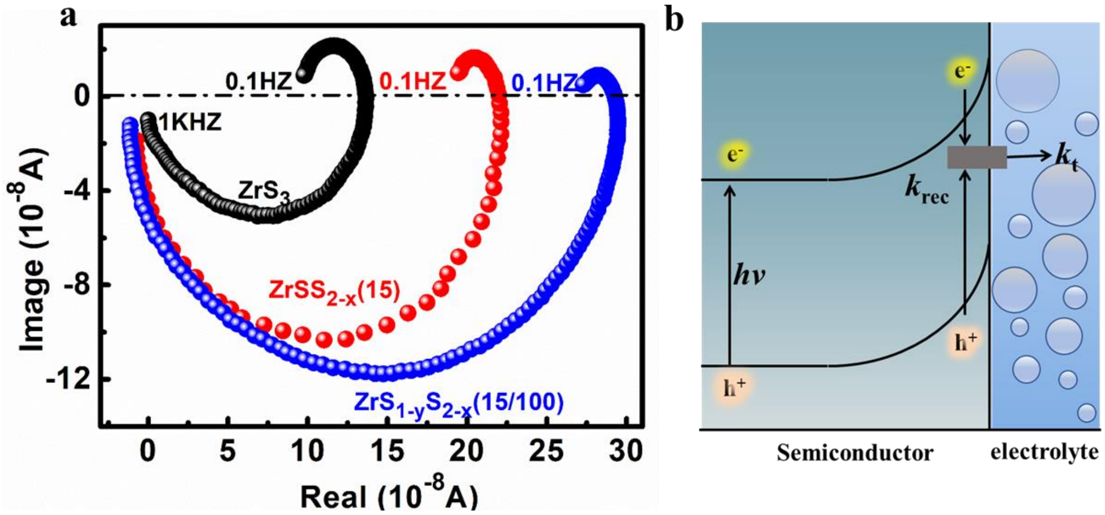

Supplementary Figure 23. Dynamics of surface charge transfer and recombination. (a) Typical experimental IMPS response measured in 0.5 M Na_2SO_4 with 0.1 M benzylamine at 0.4 V_{RHE} for the ZrS_3, $ZrSS_{2-x}$(15), and $ZrS_{1-y}S_{2-x}$(15/100) NBs. (b) Generalized reaction schematics showing the competition between charge transfer and recombination. k_t and k_{rec} are the first order rate constants for charge transfer and surface recombination, respectively.

Supplementary Notes

Supplementary Note 1. To accurately measure the NB thickness, AFM was used to obtain the height-calibrated data by imaging over 100 ZrS_3 NBs. Three representative AFM images of NBs are shown in Supplementary Figure 5a-c, which all show the ratio of width/thickness larger than 15. This suggests that all three samples display the typical characteristics of "NBs". As shown in Supplementary Figure 5d, the thickness distribution shows that the average thickness of NB is 38 nm with the peak rangeing from 25 to 45 nm.

Supplementary Note 2. To check whether any iodine is trapped within the thin film, the SEM-EDS were conducted on the sample of $ZrS_{1-y}S_{2-x}$(15/100) deposited on the FTO substrate. The SEM-EDS mapping image (Supplementary Figure 7a) and the corresponding EDS spectrum (Supplementary Figure 7b) clearly reveal that no iodine is trapped within the film. The sample on the FTO substrate was further analyzed by Raman measurement. As shown in Supplementary Figure 7c, no obvious change was observed in the Raman spectra after the deposition of $ZrS_{1-y}S_{2-x}$(15/100) on the FTO substrate, indicating that the deposition process does not affect the photocatalyst.

Supplementary Note 3. As shown in Figure 2f, g, and Supplementary Figure 7d, all the ZrS_3 NBs show the positive slope in the Mott−Schottky plots, indicating the n-type semiconducting nature of ZrS_3. The carrier density (N_d) was calculated from the slope using the following Supplementary Equation (1):[12]

$$N_{\mathrm{d}} = \frac{2}{e_0 \varepsilon \varepsilon_0} [\frac{\mathrm{d}(C^{-2})}{\mathrm{d}V}]^{-1} \quad (1)$$

where $\mathrm{d}(1/C^2)/\mathrm{d}V$, ε_0, ε, and e_0 represent the straight slope, the dielectric constant of the permittivity of vacuum, the dielectric constant of ZrS_3 (22 for ZrS_3),[13] and the electron charge, respectively. The donor densities of ZrS_3, $ZrSS_{2-x}$(15), and $ZrS_{1-y}S_{2-x}$(15/100) were calculated to be 4×10^{18}, 5.35×10^{18}, and 4.58×10^{19} cm^{-3} respectively, based on Supplementary Equation 1. The dramatic increase of donor density of $ZrS_{1-y}S_{2-x}$(15/100) is mainly due to the introduction of substantial S^{2-} vacancies during the Li treatment, which can serve as shallow donors. By extrapolating to $1/C^2 = 0$, the E_{fb}s of ZrS_3, $ZrSS_{2-x}$(15), and $ZrS_{1-y}S_{2-x}$(15/100) are estimated to -0.10, -0.11, and -0.18 V_{RHE}, which demonstrates the upward Fermi level shift of $ZrS_{1-y}S_{2-x}$(15/100).

The width of depletion region in a semiconductor (w_{d}) can be calculated using the following Supplementary Equation (2):[14, 15, 16]

$$w_{\mathrm{d}} = [\frac{2\varepsilon\varepsilon_0 \Delta\phi_{\mathrm{SC}}}{eN_{\mathrm{d}}}]^{1/2} \quad (2)$$

where $\Delta\phi_{\mathrm{SC}}$ is the potential drop in the space charge region. Since the photocatalysis occurs under the open-circuit with illumination, the $\Delta\phi_{\mathrm{SC}}$ can be evaluated by the difference of the open-circuit potential (E_{OC}) under the illumination and E_{fb}.[15] As shown in Supplementary Figure 21, the E_{OC}s of ZrS_3, $ZrSS_{2-x}$(15), and $ZrS_{1-y}S_{2-x}$(15/100) are determined to be 0.4, 0.37, and 0.39 V_{RHE}, respectively, corresponding to the $\Delta\phi_{\mathrm{SC}}$s of 0.5, 0.48, and 0.57 V_{RHE}. Therefore, the calculated depletion widths in ZrS_3, $ZrSS_{2-x}$(15), and $ZrS_{1-y}S_{2-x}$(15/100) in the solution with illumination are 55, 46, and 17 nm, respectively. This suggests a significant band bending occurring on the surface of ZrS_{1-}

$_yS_{2-x}$(15/100), which leads to the fast extraction of holes toward the surface and restricts the internal band-to-band recombination.

Supplementary Note 4. As shown in Supplementary Figure 15c, the interplanar spacings for the (010) and (100) planes of ZrS_3 are 0.37 and 0.51 nm, respectively. The exposed (001) plane consists of S_2 and S_3 atoms that can be aligned in the [100] direction (Supplementary Figure 15d), and thus the location of S_2 and S_3 can be determined by measuring the distance between the atoms in [100] direction. As a result, the atom sites in the TEM image can be specified.

Supplementary Note 5. The change in H_2O_2 concentration with time over the $ZrS_{1-y}S_{2-x}$(15/100) photocatalyst was tested to investigated the rates for formation and decomposition of H_2O_2. As shown in Supplementary Figure 17b, both rates for formation and decomposition of H_2O_2 with the presence of benzyl alcohol and benzylamine follow the zero- and first-order kinetics toward H_2O_2 concentration, respectively. The kinetic data are therefore explained by the Supplementary Equation 3:

$$[H_2O_2] = (k_f/k_d)\{1 - \exp(-k_d t)\} \quad (3)$$

where k_f (μmol h^{-1}) and k_d (h^{-1}) are the rate constants for formation and decomposition of H_2O_2, respectively. The $ZrS_{1-y}S_{2-x}$(15/100) photocatalyst shows the k_f values of 125 and 113 μmol h^{-1} with the presence of benzyl alcohol and benzylamine, respectively, and the k_d values of 0.14 and 0.16 h^{-1} with the presence of benzyl alcohol and benzylamine, respectively.

Supplementary Note 6. To extract the carrier lifetime, an open-circuit voltage decay measurement was conducted on the samples under the irradiation of simulated solar light in 0.5 M Na_2SO_4 with 0.1 M benzylamine (Supplementary Figure 21). [17, 18, 19, 20] The kinetics of charge recombination was investigated by monitoring the transient *E*oc as a function of time when turning off the illumination. Once the illumination is terminated, the accumulated electrons will be redistributed due to the charge recombination until the Fermi level reaches a new level, resulting in a gradual *E*oc decay to the original level. The carrier lifetimes (τ) at different potentials can thus be determined from the *E*oc decay using Supplementary Equation 4:[17, 18, 19, 20]

$$\tau = \frac{k_B T}{e} \left(\frac{\mathrm{d}E_{OC}}{\mathrm{d}t}\right)^{-1} \quad (4)$$

where k_B is the Boltzmann constant, T is the temperature, and e is the electron charge.

Supplementary Note 7. IMPS is a convenient way of measuring the rate constants for charge transfer and recombination. [21, 22, 23, 24] For the IMPS measurement, all the samples were illuminated by a narrow-band white LED light (100 mW cm^{-2}). As shown in Supplementary Figure 23a, the IMPS spectra show a typical semicircle in the first quadrant, representing the surface charge transfer and recombination process. The rate constants k_t and k_{rec} can be extracted from the semicircle in the first quadrant of the IMPS dates using the simple phenomenological theory (Supplementary Figure 23b).[22, 23] At the low-frequency limit, the plot intersects the real axis at the point $I_L = I_0 k_t/(k_t + k_{rec})$. I_0 is the current density corresponding to the flux of photogenerated minor carriers towards the surface. As the frequency increases, the relaxation in the concentration of photogenerated holes at the semiconductor surface is characterized by f_{max1}, which is

the frequency at the maximum of the semicircle in the first quadrant where $2\pi f_{max1} = k_t + k_{rec}$. At the high-frequency limit, the plot intersects the real axis at the point $I_H = I_0$. Thus, the k_t and k_{rec} can be calculated.

Supplementary References

1. Wang C, Zheng C, Gao G. Bulk and Monolayer ZrS_3 as Promising Anisotropic Thermoelectric Materials: A Comparative Study. *J. Phys. Chem. C* **124**, 6536-6543 (2020).
2. Shiraishi Y*, et al.* Resorcinol–formaldehyde resins as metal-free semiconductor photocatalysts for solar-to-hydrogen peroxide energy conversion. *Nat. Mater.*, (2019).
3. Wei Z, Liu M, Zhang Z, Yao W, Tan H, Zhu Y. Efficient visible-light-driven selective oxygen reduction to hydrogen peroxide by oxygen-enriched graphitic carbon nitride polymers. *Energy Environ. Sci.* **11**, 2581-2589 (2018).
4. Kofuji Y*, et al.* Carbon Nitride-Aromatic Diimide-Graphene Nanohybrids: Metal-Free Photocatalysts for Solar-to-Hydrogen Peroxide Energy Conversion with 0.2% Efficiency. *J. Am. Chem. Soc.* **138**, 10019-10025 (2016).
5. Moon G-h, Fujitsuka M, Kim S, Majima T, Wang X, Choi W. Eco-Friendly Photochemical Production of H_2O_2 through O_2 Reduction over Carbon Nitride Frameworks Incorporated with Multiple Heteroelements. *ACS Catal.* **7**, 2886-2895 (2017).
6. Shiraishi Y*, et al.* Sunlight-driven hydrogen peroxide production from water and molecular oxygen by metal-free photocatalysts. *Angew. Chem. Int. Ed. Engl.* **53**, 13454-13459 (2014).
7. Kim H-i, Kwon OS, Kim S, Choi W, Kim J-H. Harnessing low energy photons (635 nm) for the production of H_2O_2 using upconversion nanohybrid photocatalysts. *Energy Environ. Sci.* **9**, 1063-1073 (2016).
8. Mase K, Yoneda M, Yamada Y, Fukuzumi S. Seawater usable for production and consumption of hydrogen peroxide as a solar fuel. *Nat. Commun.* **7**, 11470 (2016).
9. Isaka Y, Kawase Y, Kuwahara Y, Mori K, Yamashita H. Two-Phase System Utilizing Hydrophobic Metal-Organic Frameworks (MOFs) for Photocatalytic Synthesis of Hydrogen Peroxide. *Angew. Chem. Int. Ed. Engl.* **58**, 5402-5406 (2019).

10. Xu J, *et al.* $Cd_3(C_3N_3S_3)_2$ coordination polymer/graphene nanoarchitectures for enhanced photocatalytic H_2O_2 production under visible light. *Sci. Bull.* **62**, 610-618 (2017).

11. Song H, Wei L, Chen C, Wen C, Han F. Photocatalytic production of H_2O_2 and its in situ utilization over atomic-scale Au modified MoS_2 nanosheets. *J. Catal.* **376**, 198-208 (2019).

12. Wang G, Wang Q, Lu W, Li J. Photoelectrochemical study on charge transfer properties of TiO_2-B nanowires with an application as humidity sensors. *J. Phys. Chem. B.* **110**, 22029-22034 (2006).

13. Perluzzo G, Lakhani A, Jandl S. Electrical transport measurements in a quasi-onedimensional semiconductor ZrS3. *Solid State Commun.* **35**, 301-304 (1980).

14. Zhang K, *et al.* Overcoming Charge Collection Limitation at Solid/Liquid Interface by a Controllable Crystal Deficient Overlayer. *Adv. Energy. Mater.* **7**, 1600923 (2017).

15. Giménez S, Bisquert J. *Photoelectrochemical solar fuel production*. Springer (2016).

16. Tian Z, *et al.* Novel Black $BiVO_4/TiO_{2-x}$ Photoanode with Enhanced Photon Absorption and Charge Separation for Efficient and Stable Solar Water Splitting. *Adv. Energy. Mater.* **9**, 1901287 (2019).

17. Zaban A, Greenshtein M, Bisquert J. Determination of the electron lifetime in nanocrystalline dye solar cells by open-circuit voltage decay measurements. *Chemphyschem* **4**, 859-864 (2003).

18. Bang JH, Kamat PV. Solar Cells by Design: Photoelectrochemistry of TiO_2 Nanorod Arrays Decorated with CdSe. *Adv. Funct. Mater.* **20**, 1970-1976 (2010).

19. Yang HH, Fan WG, Vaneski A, Susha AS, Teoh WY, Rogach AL. Heterojunction Engineering of CdTe and CdSe Quantum Dots on TiO_2 Nanotube Arrays: Intricate Effects of Size-Dependency and Interfacial Contact on Photoconversion Efficiencies. *Adv. Funct. Mater.* **22**, 2821-2829 (2012).

20. Tian Z, *et al.* Hydrogen plasma reduced black TiO_2B nanowires for enhanced photoelectrochemical water-splitting. *J. Power Sources* **325**, 697-705 (2016).

21. Gao Y, Hamann TW. Quantitative hole collection for photoelectrochemical water oxidation with $CuWO_4$. *Chem. Commun.* **53**, 1285-1288 (2017).
22. Cachet H, Sutter EMM. Kinetics of Water Oxidation at TiO_2 Nanotube Arrays at Different pH Domains Investigated by Electrochemical and Light-Modulated Impedance Spectroscopy. *J. Phys. Chem. C* **119**, 25548-25558 (2015).
23. Peter LM. Energetics and kinetics of light-driven oxygen evolution at semiconductor electrodes: the example of hematite. *J. Solid State Electrochem.* **17**, 315-326 (2013).
24. Ponomarev EA, Peter LM. A Comparison of Intensity-Modulated Photocurrent Spectroscopy and Photoelectrochemical Impedance Spectroscopy in a Study of Photoelectrochemical Hydrogen Evolution at P-Inp. *J. Electroanal. Chem.* **397**, 45-52 (1995).
